# Alkyd resins produced from bio-based resources for more sustainable and environmentally friendly coating applications

**DOI:** 10.55730/1300-0527.3511

**Published:** 2022-10-08

**Authors:** Cemil DIZMAN, Elif CERRAHOĞLU KAÇAKGİL

**Affiliations:** İzel Kimya Research and Development Center, Dilovası, Kocaeli, Turkey

**Keywords:** **A**lkyds, bio-based polymers, coating, condensation polymerization, paint industry, sustainability

## Abstract

Recently, due to the depletion of natural resources and raising environmental and economic concerns regarding petroleum derivatives, the creation of novel ecologically friendly and sustainable materials made from bio-based and renewable resources is gaining popularity. Alkyd resins are synthetic resins in which both renewable (fatty acids, glycerol, oil, etc.) and nonrenewable (maleic anhydride, pentaerythritol, phthalic anhydride, etc.) raw materials are used in their production. Due to their superior performance (good aging, greater weather resistance and high heat resistance, outstanding gloss, etc.) over other resins, easy application, low cost, and varied use, in the coating and paint industries, they are commonly used. This review covers the studies on bio-based monomers used instead of nonrenewable ones in the production of alkyds. The effects of substituted bio-based monomers on the final properties (adhesiveness, drying times, hardness, tackiness, etc.) of produced alkyds and coatings are also discussed in detail.

## 1. Introduction

In recent years, the principle of “sustainability” has been one of the biggest drivers and requirements of the chemical industry due to the scarcity of traditional raw materials, high oil prices, waste disposal, climate change and environmental pollution concerns [1–6].

**Figure 1:**
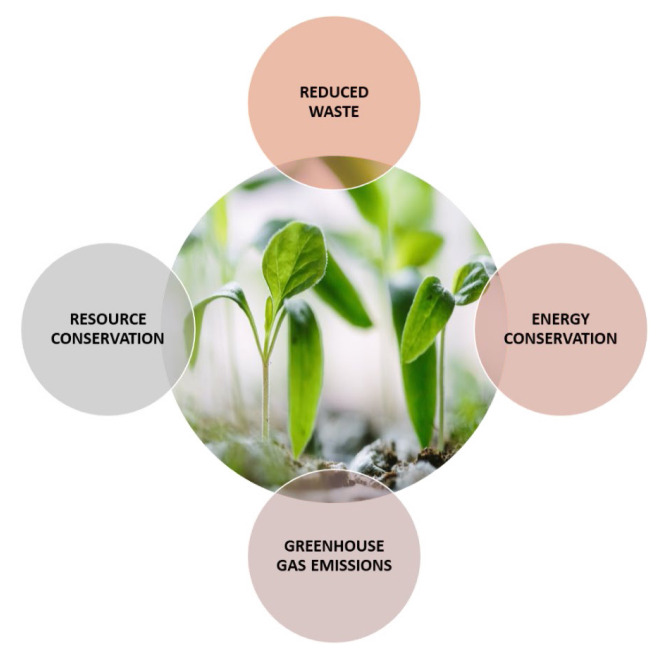
Scheme of some sustainability gains.

For the transition to a bio-based circular economy; there are motivations such as the safety of processes and products, the desire to reduce overreliance on fossil fuel imports, the need to reduce atmospheric greenhouse gas (GHG) emissions, the design of chemicals for low-cost materials and energy efficient recycling (Figure 1) [7,8].

Besides these motivations, there are several studies for understanding the concept of sustainable bio-based economy with a view of economic, politic and social [9–12]. Plastics have an important place in the modern economy as an indispensable part of daily life. Plastics are made from polymers and their use is increasing day by day [13,14]. Polymers, which are versatile materials, are largely prepared from petroleum-based feedstock [15–17]. Concepts such as a lower ‘carbon footprint’ and ‘sustainable production’ are also increasingly used in the polymer industry and are increasingly being incorporated into the other industries, too. In addition, the move to more sustainable products can open up opportunities to improve efficiency and product performance and to produce products with new features [18–21].

Generally, the products used for the coatings industry are generally of polymer origin, and this industry is trying to reduce its reliance on petrochemicals while prioritizing the use of natural oils as environmentally friendly starting materials. The surface coating industries are dominated by the use of oil-modified polyesters or alkyds as solvent-based binders [22].

Alkyd resins are commonly employed as binders, adhesives, and plasticizers in surface coatings. They are made by combining polyols and anhydrides or acids such as maleic anhydride, phthalic anhydride, and isophthalic acid with fatty or oily fatty acids in a condensation polymerization reaction. Alkyd is interesting because it has the least expensive resins of the coating materials and has fewer film defects during application [23–25].

**Figure 2:**
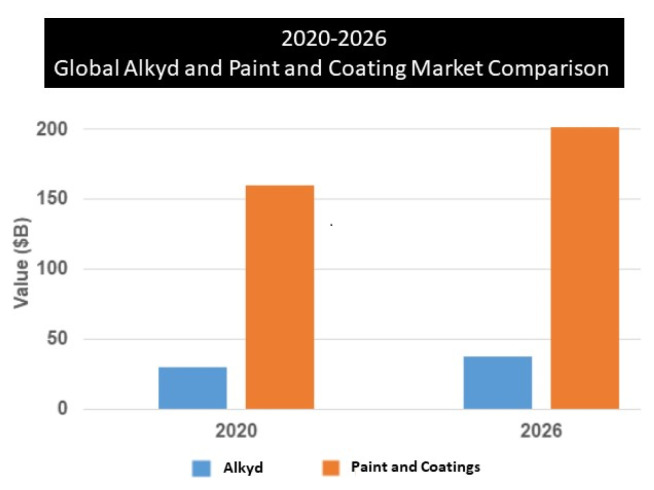
Alkyds in paint and coating market, 2020–2026, in USD billion.

When we look at the commercial volume of the paint and coating industry, it is seen that it is around $160 billion in 2020 and is increasing day by day with a 4% growth rate per year. In this market, which is thought to be approximately $202 billion in 2026, the alkyd resin market is estimated to reach $37.56 billion (Figure 2) [26,27].

**Figure 3:**
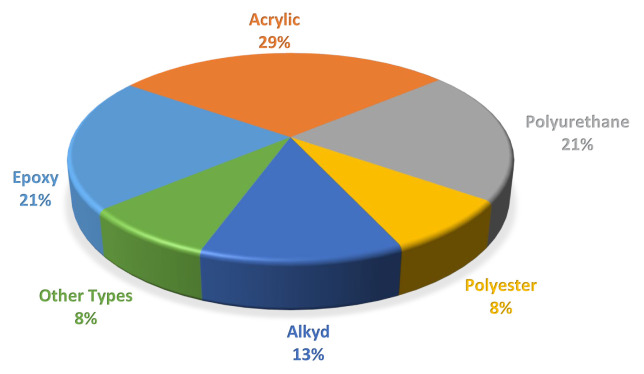
Resins in paints and coatings market, revenue (%), by type in 2019 [28].

Figure 3 depicts the global distribution of various resins (alkyd, acrylic, epoxy, polyester, polyurethane, and others) used for various applications in the paint and coating market in 2019.

**Figure 4:**
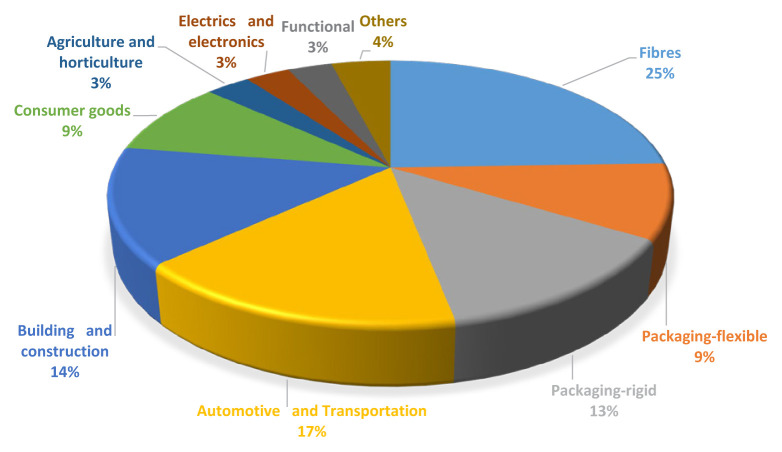
Bio-based resin market, volume share (%), by applications in 2020.

Bio-based economy is expanding in use of polymers. In a study conducted in 2020, the usage volumes of bio-based resins were examined according to their application areas in order to evaluate the market trend (Figure 4) [29].

Surface coatings exhibiting excellent hardness, adhesion and flexibility can be obtained with bio-based renewable resins [30–33]. Because of their low cost and environmental friendliness, renewable and bio-based oils are commonly employed in polymer preparation [34–37].

Vegetable oils are intriguing because they are relatively straightforward to separate and include a range of functional groups that can be used to derivatize chemicals. Linseed oil, for example, has a high amount of unsaturation, allowing it to be used as a surface coating, but palm oil has a low level of unsaturation. Because vegetable oil triglycerides have long, highly flexible aliphatic chains, crosslinked networks obtained from unsaturated vegetable oils through autooxidation, a free radical chain reaction process that produces a variety of crosslinking reactions via free radical coupling reactions may be too soft for certain applications [38–41].

Iodine value measures the unsaturation in oils. The higher degree of unsaturation implies that the oil has sufficient double bonds for crosslinking by the help of siccatives. Iodine value and double bond contents of some edible and nonedible oils are shown in Table 1 [42–44].

**Figure 5:**
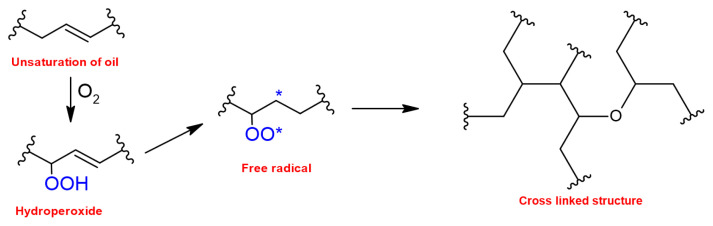
Autoxidation process of alkyd resins.

Oils and fatty acids with different chemical structures, whose chemical structure and natural state can be seen in Table 2, cause the obtained alkyd resin to have different properties. The chemical structures of the oil and fatty acids utilized in alkyd synthesis also dictate how and where alkyds can be used. Oils that dry out, such as, castor, linseed, soybean, sunflower, etc., are generally used in preparation of long and medium oil alkyds that can be cured with siccatives by their autooxidizable double bonds as illustrated in Figure 5 [45].

Nondrying oil and fatty acids such as, coconut oil, palm oil, lauric acid, etc., do not in its molecular structure contain double bonds and used in preparation of short oil alkyds that can be cured with another component such as, isocyanate or melamine [46–48].

Linseed, soybean, sunflower and tall oil have similar chemical structures. However, their chemical compositions are different. Linseed oil contains the triply unsaturated α-linolenic acid (49.85%) and the doubly unsaturated linoleic acid (16.67%). Alkyds produced from linseed oil dry fast because they contain highly unsaturated double bonds. Soybean oil contains less triunsaturated α-linolenic acid (7.65%) and the doubly unsaturated linoleic acid (52.58%), which causes alkyds made from them to have slower drying rates than alkyds made from linseed oil [49].

When considered in general today, the renewability content of solvent-based alkyd resins is around 50%–60% by weight. However, this ratio is higher in long oil alkyds. As a rule, it can be said that this ratio increases as the oil content increases. The main problem with producing more than 80% by weight renewable alkyd resins is the absence of large-scale renewable, diacids or anhydride substitutes for the anhydride derivatives used. Furthermore, there are no commercially available substantial quantities of renewable benzoic acid alternatives for short oil alkyd syntheses. Several attempts to develop renewable and hard dicarboxylic acids have also been made. However, all signs point to the possibility of developing renewable alkyd resins that are 80%–95% by weight in the near future [22,47,50]. In addition, environmentally friendly technologies with high solids, hyperbranched, water-based and UV-curable are also currently being researched [45,51].

The raw materials used in the production of bio-based alkyd resins were investigated in this study, which is a very valuable and important subject for our present and future, and the studies on the properties of the resins obtained from these raw materials were examined in detail.

## 2. Synthesis of alkyd resins

First produced in the 1920s, alkyd resins, part of a large class of modified condensation polymers known as polyesters, began to be produced on a commercial scale in 1933.

**Figure 6:**
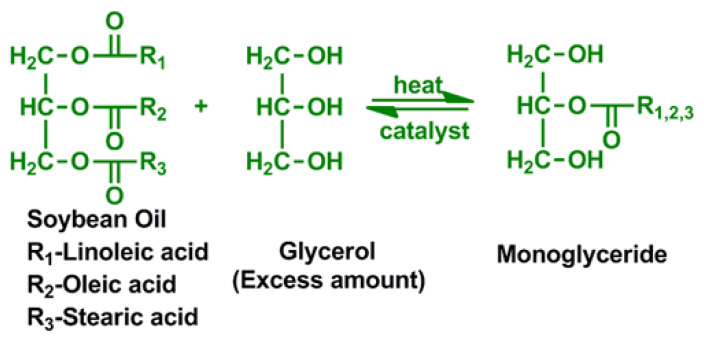
Transesterification to form monoglyceride.

Glycerol is generally used as the polyol for the monoglyceride process, which is the most common method in alkyd resin synthesis. This process starts with the transesterification step. The oil reacts with the glycerol. At temperatures ranging from 230 °C to 250 °C, this process is usually done with a catalyst. It usually uses glycerol as the polyol for the monoglyceride process, which is the most common method in alkyd resin synthesis [52–56]. The schematic representation of transesterification of glycerol and an oil sample is given in Figure 6.

**Figure 7:**
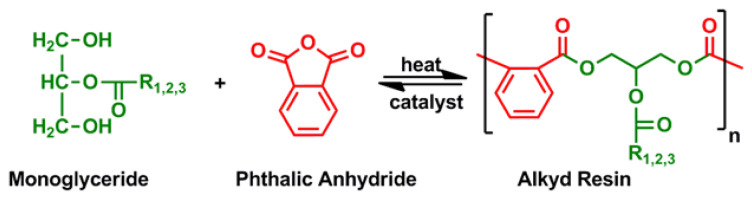
Monoglyceride and phthalic anhydride polymerize to form an alkyd resin.

The next step involves the immediate esterification of dibasic acid or anhydrides (isophthalic acid phthalic anhydride, etc.) and monoglyceride as shown in Figure 7.

**Figure 8:**
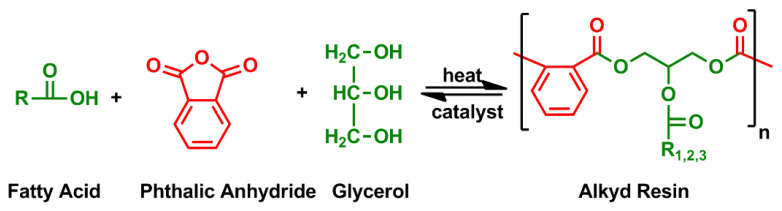
Polymerization of alkyd resin.

In case of using fatty acid instead of oil at the beginning, alkyd synthesis is performed in one-step as shown in Figure 8. In this method, fatty acid, dibasic acid, polyol (glycerol or pentaerythritol) and other monomers (benzoic acid, gum rosin, phenolic resin, etc.) for modifications are put together into a reactor and at high temperatures, both aromatic and aliphatic acids are esterified simultaneously [57].

## 3. Synthesis and types of alkyds produced by conventional methods

As mentioned in Section 2, alkyd resins are polyester-based compounds that have been treated with oil or fatty acids. The performance attributes of the manufactured alkyd resin are affected by the monomers used [22,47,50,58]. In addition, the oil length in the alkyd formulation is one of the most crucial determinants of the final properties and usage areas of the product.

### 3.1. Long-medium-short oil alkyd resins

There are three different types of alkyd resins: short, medium and long oil resins according to their oil lengths. Short, medium and long alkyd resins contain approximately 30%, 50%, and 60% oil by weight, respectively [59].

#### 3.1.1. Long oil alkyd resins

Drying and semidrying oils are typically used to make long oil alkyds. Soy, linseed and sunflower oils are the most commonly used oil. Glycerol and/or pentaerythritol are generally preferred as a polyol source. Long oil resins are extremely soluble in aliphatic solvents with a low odor. Thanks to their properties, they are used in architectural and maintenance areas, such as primers, brush enamels, and marine paints. Their properties also vary depending on the oil in which they are used, but they generally have reasonable drying times and good yellowing resistance. For example, this group of resins, which has good drying properties when using linseed oil, has low UV yellowing resistance and limits primer and topcoat applications to dark colors. In the case of using safflower oil, better drying and yellowing resistance can be obtained compared to linseed oil, but its price remains quite high. Long oil alkyd resins produced using sunflower oil have properties between soybean and safflower [60–62]. Table 3 shows some application samples for long oil alkyd resins.

#### 3.1.2. Medium oil alkyd resins

Medium oil alkyd resins are a versatile group among other alkyd groups. Because of their solubility in low boiling aliphatic solvents, they have a wide range of uses. They are generally used for anticorrosion primers and general paint applications in outdoor environments, but the application properties vary according to the type of oil used in their production. For example, when linseed and soybean oil are used, it finds use in automotive refinishing paints and application enamels. For medium oil alkyd resins, some application samples were given in Table 4.

#### 3.1.3. Short oil alkyd resins

Unmodified and fast drying short oil alkyds are usually made from soy, linseed and hydrolyzed castor oil. As with other alkyd groups, its properties and application areas vary according to the type of oil used. For short oil alkyd resins, some application samples were given in Table 5.

Short oil alkyd resins made from soybean and dehydrolyzed castor oil dry more slowly than long oil alkyd resins, although they can be utilized in air-drying systems. Linseed oil-based enamels are utilized in a variety of applications, including general-purpose industrial air-drying enamels and automotive repair enamels. Alkyds made from coconut oil have a very high resistance to the external environment, while varnishes also show very good film properties. Most of the styrenated alkyds are among the short oil alkyds. In addition to its quick drying advantage, its poor recoating properties limit its use. For this reason, they are generally used as rigid single coat varnishes [15].

### 3.2. Urethane and chain stopped (rapid) alkyd resins

Alkyd resins are modified in various ways for applicability and use. These modifications are made by adding various additives or by reacting with different chemicals in order to obtain resins with the traits that are desired. Alkyd resins are generally modified with vinyl, methyl methacrylate, phenolic compounds, urethane, styrene, benzoic acid and derivatives, epoxy and isocyanates [22,47,50,58,59,80].

#### 3.2.1. Urethane alkyd resins

Urethane alkyds can be defined as alkyd resins containing diisocyanates (usually TDI). The two main advantages of these resins are good abrasion resistance and better hydrolysis resistance compared to standard alkyd coatings. The disadvantage is that the color of the films darkens over time and is a little more expensive than other alkyds. Its use in light-colored paints is limited due to its low yellowing resistance [24,81]. For urethane alkyd resins, some application samples were given in Table 6.

#### 3.2.2. Chain-stopped (rapid) alkyd resins

There are many alkyd derivatives obtained on the basis of the type of oil, other components that make up these oils, other raw materials used and reaction pathways [24]. Chain-stopped alkyds are fast drying alkyds developed for industrial primer and enamel applications [86,87]. These resins are commonly used in air-drying varnishes and industrial oven varnishes. Their fast drying times, great durability, strong hiding power, and high gloss qualities make them popular in the automotive industry. Chain-stopped alkyd resins are used with pigments, extenders, binders, various additives suspended in a solvent that carries the suspension to a substrate, and then the solvent evaporates to form a dry film [88]. For chain-stopped alkyd resins, some application samples were given in Table 7.

### 3.3. Polyurethane alkyd resins

The polyaddition reaction with alcohols and isocyanates such as toluene diisocyanate (TDI) and hexamethylene diisocyanate produces polyurethanes (HMDI). It is used for the synthesis of polyurethane as well as aliphatic and aromatic isocyanates, vegetable oil amide diols and polyols. Vegetable oil-based monomers and polymers with having hydroxyl end functional groups such as amide diols, alkyds, and metal/metalloid have been frequently used in the synthesis of polyurethane coatings. Vegetable oil polyurethanes contain functional groups such as vinyl, amide, ester as well as urethane parts. Its functional groups increase impact resistance, adhesion, flexibility and scratch hardness, and provide coatings with better chemical resistance. The percentage of degradable content in vegetable oil-based polyurethanes varies depending on the isocyanate or polyol. The advantages of polyurethane coatings are that they usually cure or dry at ambient temperatures [7]. For polyurethane alkyd resins, some application samples were given in Table 8.

**Figure 9:**
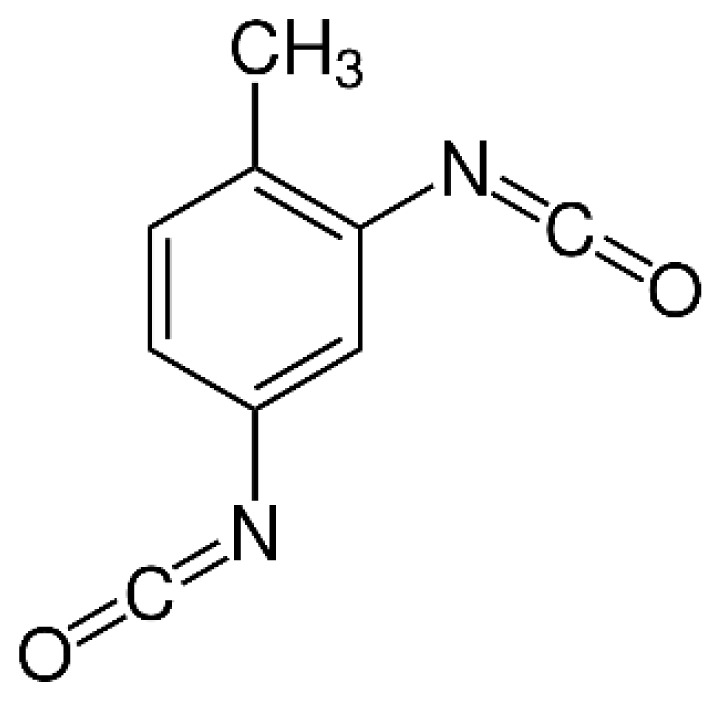
TDI molecular structure.

The discrepancy in activity of the two isocyanate groups is exploited in TDI-based polyurethane (the molecular structure of TDI is shown in Figure 9). Polyurethane coatings typically require multiple steps to cure. These include evaporation of the solvent in the structure, the reaction of free isocyanates with moisture in the environment, and autooxidative crosslinking in the backbone of vegetable oil derivatives [7,96]. In addition, polyurethanes prepared from polyols obtained with lactic acid and epoxidized soybean oil generally have lower glass transition temperatures (Tg) (30 °C) than those obtained with vegetable oil, compared to higher glass transition temperature Tg values (31–96 °C) [97].

## 4. Nonrenewable and renewable monomers used for the synthesis of alkyds

### 4.1. Nonrenewable monomers

**Figure 10:**
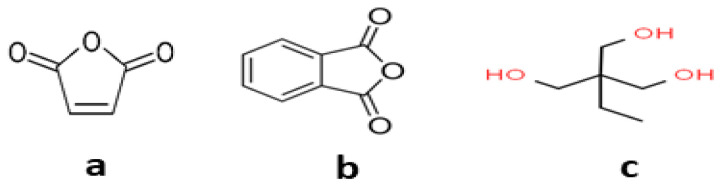
Structures of some nonrenewable monomers a) maleic anhydride, b) phthalic anhydride, and c) trimethylolpropane.

When looking at the researches in the literature, it is clear that petroleum-based monomers are commonly employed in the production of alkyd resin. Table 9 summarizes the raw ingredients employed in several investigations as well as their quantities. Structures of some of the nonrenewable monomers were given in Figure 10.

In a study, alkyd resin was synthesized using neem (Azadirachta indica) seed oil and its anticorrosive coating applications were investigated. Using a soxhlet apparatus and petroleum ether as a solvent, the oil in the structure of neem seeds was extracted. The extracted oil was prepared utilizing a 10% ethyl acetate and 90% hexane solvent mixture and column chromatography on silica gel 60–120 mesh. The alkyd resin was made using the alcoholysis-polyesterification technique. In this two-step synthesis, the first monoglyceride formation was achieved. In the second stage, polyesterification of alkyd resins was completed with the addition of anhydride. Four different resins (Resin A, B, C, D) were tested by varying the maleic acid and phthalic acid ratios. As a consequence of the investigation, the average molecular weight (Mw) was stated as 2443, 2649.5, 2745, and 5596.5 g/mol, respectively. Curing times were found to be 8, 7, 6, and 4 h in the same order at 210 °C. It was discovered that increasing the amount of maleic anhydride in the formulation reduced the curing time of alkyd resins. In addition, pencil hardness values are HB, HB, 2B, 2B for each formulation, respectively; luminance values at 60 °C were determined as 85, 64.4, 48.2, and 36.3. The thermal degradation of them after analysis exhibited a two-step weight loss behavior [98]. The first stage weight loss for resins A, B, C, and D was observed at 150–160 °C, whereas the second stage weight loss was observed at 250 °C, 288 °C, 305 °C, and 316 °C, respectively. Accordingly, TGA thermograms showed that the thermal stability of them was quite good. Figure S1 shows the DSC and TGA thermograms, while Table S1 summarizes the other findings.

Sacha inchi oil, pentaerythritol, trimethylolpropane were used in the alkyd resin synthesis study by Obregón et al. For the resins hard drying times and pencil hardness values were found as 182, 132, 255, 370, 390, 150, 240; 4H, 4H, 4H, 4H, 2H, 2H, 4H, respectively. In addition, the acid values are 15.7, 4.7, 19.2, 17.6, 17.2, 9.2, 17.7; Gardner viscosity values are Z6–Z7, Z7–Z8, > Z10, Z7–Z8, Z6–Z7, Z6–Z7, Z5–Z6 as given in Table S2. Thermogravimetric analyzes of the prepared alkyd resins were analyzed, the thermograms were given in Figure S2. The quantity of TMP has a less weight loss during heating at 244–360 °C. Nevertheless, at above 500 °C, only the SM-MP/PE (75/25) sample has a higher thermal stability with the lowest weight. Relating to the DTA analysis, it was determined that the thermal degradation of alkyd resins was occurred at about 470 °C [99].

In the study conducted by Ekpa and Isaac, three different groups of alkyd samples with oil percentage of 40%, 50%, and 60% as short, medium, and long oil, respectively, were synthesized and analyzed. The examples given in Table 8 are prepared as short oil alkyd resin of refined melon seed oil, short oil alkyd resin of crude melon seed oil, medium oil alkyd of raw melon seed oil and long oil alkyd resin of raw melon seed oil. PbO is used as the catalyzer. To evaluate the performance properties of alkyd resins, alkyd samples were formulated as white gloss paint. Drying times of melon seed oil alkyd and standard soy alkyd paint samples formulated without lead were determined as 780, 660, 1260, 1442, and 660 min for PREMESAR 1, PCMESAR 2, PCMESAR 3, PCMESAR 4 and standard soy alkyd paint (SAP). In addition, indoor hard drying results of standard soy alkyd paint samples were formulated without the use of alkyd paint and lead drier were determined as 900, 810, 1440, 1800, and 750 min for PREMESAR 1, PCMESAR 2, PCMESAR 3, PCMESAR 4 and standard soy alkyd paint. (SAP), respectively. Outdoor drying times obtained with lead octoate were 420, 390, 510, 580, and 360; closed drying times were determined as 465, 420, 540, 635, and 420 min (Example PREMESAR 1–40% with refined melon seed oil; Sample PCMESAR 2–40% with crude melon seed oil; Sample PCMESAR 3–50% with crude melon seed oil; Sample PCMESAR 4–60% with crude melon seed oil). In addition to these studies, pencil hardness values of alkyd paints made with melon seed oil were determined as 4H, 4H, 3H, 3H, 5H for scratches and cavities; 5H, 5H, 4H, 4H, 6H. Table S3 summarizes the findings [60].

Dhakite and Burande used coconut oil, resin, glycerol, and maleic anhydride to make NAR-I alkyd resin. Its physicochemical properties were found to have an acid value of 30.89, a dark brown color, a viscosity of 238 s, and an oxiraneoxygen value of 13.2. According to the IR spectrum they examined, the peak at 3444.36 cm^−1^ showed the presence of OH groups in the alkyd molecule. The peaks at 2888.29 cm^−1^ revealed the C–H stretch group. The peak at 1726.11 cm^−1^ was due to the aromatic C=C stretch. C-O stretching created the peak at 1160 cm^−1^, while C=C stretching caused the peak at 1640 cm^−1^. C-H outside of the plane-bending mode in the combination produced the peak at 762.5 cm^−1^. Because of the physicochemical analysis of the new alkyd resin, the HLB (hydrophobic lipophilic balance) ratio was determined to be between 13 and 14. The high acid value obtained allowed the resin to be neutralized and thus transformed into a water-soluble product. In addition, the molecular weight of the samples was obtained in the range of 4500 g/mol to 5000 g/mol, which is ideal for use as a polymeric surfactant [100].

According to Ikhazuagbe et al., alkyd resins were synthesized by the monoglyceride method using rubber seed oil (RSO) and soybean oil (SBO). Alkyd samples obtained using different ratios of RSO and SBO were characterized for comparison purposes and procedure for synthesizing alkyd resin given in Figure S3. The drying properties (click time and tackiness) of the alkyd resin synthesized using 100% RSO oil did not improve significantly as the mixing ratio of SBO in RSO increased. The test results of the resins are given in Table S4. Nonadhesion and drying times are estimated as 129, 131, 132, 128, 130, 132, 133 min; 238, 241, 237, 235, 237, 239, 343 min. Alkyds based on RSO/SBO have iodine values of 157.90/142.00, 152.44, 149.90, 143.16, 142.26, and 142.41 gI_2_/100 g; it has saponification values of 216.21/228.61, 203.67, 171.04, 161.97, 142.89 and 135.62 mg/KOH [79].

Salata et al. synthesized long oil alkyds resin (LOA) using linseed oil. Then, alkoxysilane modified linseed oil alkyds (TESLOA) were obtained by modifying it with 3-(triethoxysilyl) propyl isocyanate (TESPIC). Viscosities of resins obtained by adding 10%, 20%, 30%, and 40% TESPIC to long oil alkyd were measured as 4900 ± 200, 40,400 ± 900, 55,400 ± 2200 cP and gel, respectively. The glass transition temperatures of the uncured states of TESLOA0, TESLOA10, TESLOA20, TESLOA30 and TESLOA40 modified resins are 6.4 ± 1.8, 7.5 ± 1.2, 5.9 ± 1.1, 7.4 ± 1.7, 4.0 ± 1.5 °C; and the cured states were determined as 25 ± 3.37 ± 4.35 ± 4.31 ± 2 °C and 32 ± 6 °C. The results also showed a higher crosslink density for resins made with TESLOA. TESLOA10 had the most raised high crosslink value of density with 0.081 ± 0.013 moles of crosslinks per mole of alkyd, while a slight reduce was observed ranging from TESLOA10 to TESLOA40. However, all samples had higher crosslink values compared to the control without TESPIC. When the coating performances were examined, 20° gloss 101.9 ± 3.1, 104.9 ± 1.2, 103.6 ± 3.0, 105.1 ± 1.1, 104.7 ± 1.8; 60° gloss 111.3 ± 0.6, 111.3 ± 0.3, 111.0 ± 0.4, 112.2 ± 0.5, 112.9 ± 0.3; contact angle 80°; 7 ± 1.1, 101.1 ± 0.6, 101.5 ± 0.8, 101.7 ± 0.5, 100.8 ± 0.4; pencil hardness values were 4B, 4B, 3B, 3B, 2B; pendulum hardness values were determined as 6, 4, 5, 5, 6 and given in Table S5 [101].

Xu et al. synthesized an acrylated alkyd resin based on tung oil, isobornyl acrylate and phthalic anhydride. Tung oil-based acrylated alkyd resin characteristics as physicochemical were examined. TG and DSC curves were given in Figure S4. Acid values for alkyd resins containing 0, 10, 20, 25, 30 (wt%) IBOA, respectively, are 17.4, 18.9, 20.2, 19.8, 20.3 mg KOH/g; viscosity values 7.94, 8.21, 8.09, 8.16, 8.44 Pa s; hydroxyl values were determined as 74.1, 74.4, 76.5, 75.9, 76.7 mg KOH/g. Also, the pencil hardness values are H, H, 2H, 3H, 3H, respectively; water absorption (%) 3.54, 2.38, 1.06, 0.99, 0.89; surface roughness (nm) 1.02, 2.19, 3.92, 4.39, 5.26; breaking tensile strength (MPa) was measured as 6.13, 8.22, 9.28, 10.09, 10.89 (Table S6) [102].

Flores et al. used a two-step alcoholysis-esterification course using sacha inchi and linseed oil (control) and varied ratios of glycerol, phthalic and maleic anhydride to make medium and short oil alkyds. Monomer ratios for SS-0%, LS-0%, SS-2%, SS-4, SM-0%, LM-0%, SM-2, SM-4% are given in Table 1 and as well as the values in Table S7. The esterification continued until the acid value was close to or less than 10 mg KOH/g, according to the physical characterization follow-up. The achieved values for short oil alkyd resins range from 9.9 mg KOH/g to 11.7 mg KOH/g. Medium oil alkyd resins achieved values ranging from 8.3 mg KOH/g to 9.6 mg KOH/g. Gardner viscosity values are Z5, Z4, Z5, Z4, Z10, Z10, Z10, Z9; and colors are 12, 13, 7, 7, 10, 3, 3, 3. Drying time values are 160, 150, 130, 120, 240, 230, 180, 160 min; and pencil hardness values are 4H, 4H, 2H, 2H, 2H, 2H, HB, HB. Thermal degradation temperatures for short and medium oil resins in sacha inchi oil are 220, 247, 250, 276, 296, 305 for 10%; 50% for 341, 341, 342, 357, 360, 370; 402, 404, 410, 421, 441, 448 for 90%. TGA thermograms of SS-0%, LS-0%, SM-0%, LM-0%, SM-0%, SM-2% and SM-4% are given in Figure S5 [103].

Elba and colleagues obtained an alkyd resin with soybean oil, glycerin, and zirconium octoate (zirconium 2-ethyl hexanoate). The alkyd resin was produced by polycondensing alcoholysis products with phthalic anhydride at 250 °C. In their characterization study with FT-IR, the characteristic peaks of the alkyd resin in the spectrum are 3472.55 cm^−1^ for OH stretching vibration, 3008.8 cm^−1^ for olefinic CH stretching vibration, 2925.65 and 2854.38 cm^−1^ for CH aliphatic stretching vibration, 1735.1 cm^−1^ for the C=O stretching frequency of the ester, 1599.45 and 1580.28 cm^−1^ for the C=C stretching frequency of the alkene and aromatic band, 1489.18 and 1377.7 cm^−1^ for the symmetrical and asymmetrical bending of the methyl groups, 1280.77 and aliphatic and 1041.64 cm^−1^ for C-O-C stretching vibrations coupled with aromatic moiety, 742.65 and 705.98 cm^−1^ for out-of-plane aromatic CH bending vibrations. Set-to-touch (min), adhesion %, hardness (s) and nonstick (min) values were measured as 75, 14, 360, 100, respectively [104].

Abdullahi et al. formulated three grades of alkyd resins given in Table S8. The iodine value of the synthesized Luffa aegyptiaca seed oil-modified alkyd resin (LASOMAR) was 72.1 ± 0.74, 78.1 ± 1.05, and 83.2 ± 1.0 g _I2_/100 g for samples I, II, and III, respectively, according to physicochemical characterization. The corresponding saponification values were 205.2, 295.8, and 332.5 mg/KOH g, respectively. Acid values were found as 6.34, 10.37, 7.85 mg KOH/g; densities as 1.68, 1.04, 1.51 g/cm^3^; specific gravities as 1.91, 1.18, 1.72; refractive indexes at 30 ºC as 1.500, 1.494, 1.491 [105]. Results are given in Table S8.

Uzoh et al. synthesized alkyd resins with different formulations using gmelina seed oil as the test results are given in Table S9. Acid numbers of diluted alkyd resins were calculated as 10.80, 12.09, 13.01, 14.23 mg KOH/g. The chemical film properties for water resistance were determined and given in Table S9 [106]. When the literature studies and industrial productions are examined, it is seen that petroleum-based raw materials are widely employed in the synthesis of alkyd resins with different properties. However, it is expected that these petroleum-based raw materials used will harm the environment, oil prices will increase and the supply of nonrenewable resources will become more difficult today and in the future. Phthalic anhydride widely takes part in reactions as a chemical intermediate monomer, especially in the coatings and plastics industry. Exposure and damage to phthalic anhydride monomer may occur during work. In humans, phthalic anhydride exposure has acute (short-term) repercussions such as irritation of the eyes, respiratory tract, and skin. In working persons, phthalic anhydride can have chronic (long-term) consequences. Bronchitis, injury to the skin and mucous membranes of the respiratory system, rhinitis, and conjunctivitis are some of the side effects. In addition, phthalate esters have been defined in the literature as chemicals that harm the environment [60,107,108]. It has been determined in the literature that maleic anhydride is a systemic toxic substance related to human health, aquatic life and environmental effects. For oral exposure, the acceptable daily intake (ADI) for maleic anhydride is 0.10 mg/kg/day. Maleic anhydride has a reportable quantity (RQ) of 100 [109].

Maleic anhydride has been reported to irritate the skin in a number of studies. Two workers exposed to contaminated clothing experienced skin burns and irritation, which became more acute while showering. Maleic anhydride in powder or vapor form has been related to conjunctivitis, inflammation and edema of the eyelids, intense weeping, and photophobia [110]. The other common monomer, pentaerythritol, may cause eye irritation, skin irritation and gastrointestinal irritation. The toxicological properties of this substance have not been fully studied. However, it may cause respiratory tract irritation [111]. The use of sustainable raw materials that do not harm the environment in the production of alkyd resin is a very important research and study area due to its disadvantages such as the damages it has or may have the difficulty of supply and waste disposal. As environmental and safety regulations become increasingly stringent, coating manufacturers are accelerating their approach to safer and greener products. Consumer preference for low-emission and greener products is largely driven by concerns not only about the environment and the planet, but also about their own health. Therefore, consumers have an increasing preference to purchase products containing bio-based materials, which are perceived as healthier and more environmentally friendly today. Bio-based resins also have additional advantages. These include coatings exhibiting excellent hardness, adhesion and flexibility, extremely fast drying times of prepared coatings, flexible and hard surface coatings and self-crosslinking ability after solvent evaporation through the oxidation process [112–114].

In addition, according to the results of a 2019 (2016?) study in the European Coatings Journal, the use of bio-based monomers in coating applications is more important than it was five years ago, according to approximately 77% of the respondents [115].

### 4.2. Renewable monomers

**Figure 11:**
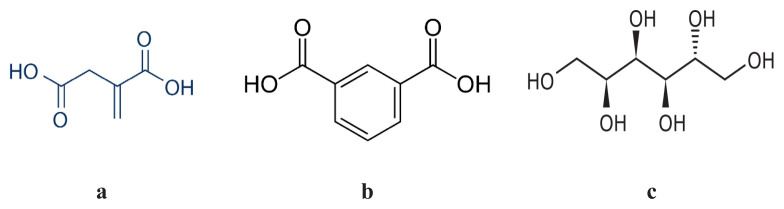
Structures of some renewable monomers a) itaconic acid, b) isophthalic acid, c) sorbitol.

Today, despite some limitations such as viscosity and thermal stability, the use and research of bio-based raw materials instead of petroleum-based raw materials is increasing in alkyd resin synthesis and production [115]. Structures of some renewable monomers are given in Figure 11.

Raw material ratios of alkyd resins synthesized with some bio-based monomers in the literature are given in Table 10. In the first stage, monoglyceride was obtained by reaction between linseed oil and glycerin at a ratio of 1:2 with the help of a catalyst. Then, the reaction mixture was kept constant at 170–180 °C and 1 mole of itaconic acid was inset to the reaction medium. For the epoxidation of the synthesized alkyd resin (EA), conversion to the oxirane ring was performed towards of acetic acid, Amberlite IR-120H and hydrogen peroxide. H_2_O_2_ was also added to the reaction medium to initiate this conversion.

APTMS content of 10, 20, 30, and 40 moles was added to the EA resin backbone (EA1, EA2, EA3, EA4). Brushing and mechanical tests of resin coatings were examined and the results are given in Table S10. The brightness (60°) was 95.24, 98.42, 101.2, 102.3, 108.20; the pencil hardness was 3H, 3H, 4H, 4H, 5H; and the scratch hardness was 1950, 2000, 2500, 2100, 2250, according to the data. According to the results of thermogravimetric analysis, Td5 (°C) was 213.29, 228.18, 233.93, 226.92, 246.97; Td30 (°C) was determined as 347.25, 351.78, 356.38, 359.32, 371.12. Also TGA and DSC thermograms were given in Figure S6.

The coating visuals before and after the corrosion test were shown in Figure S7. Figure S7 clearly shows that corrosion spreading along the crosscut is more significant in the conventional alkyd coating than in the APTMS modified coatings [50].

In another study conducted by Koning et al., the succinimide-based alkyd was prepared in a two-step procedure. To begin, a prepolymer was created at 230 °C using 367 g of soybean oil fatty acids, 234 g of bio based pentaerythritol, 200 g of imide, and 193 g of petroleum-based phthalic anhydride. Then 38 g of succinic acid was added. Resin 2 is glycine-phenylalanine citrimide; Resin 3 is phthalic anhydride-glycine succinimide; Resin 4 is a phthalic anhydride-phenylalanine succinimide resin. Bio-based imide building blocks used for the synthesis of alkyd resins are given in Figure S8. Fifteen-day König hardness (s) values for resin 1, 2, and 3 were 76, 83, 89; the drying adhesion time (h: min) was found to be 0:34, 0:31, 1:38 (Table S11). According to the results of their study with a renewable content of more than 80% by weight, it was determined that imide-containing building blocks may could be included in alkyd resins and fast-drying and hardness-enhanced coatings can be obtained [116].

Uzo et al. used the formulation given in Table S12 in their study. They studied alkyd resins of medium oil length, the resin oil content ranging from 45% to 55% of the total resin content. They determined that the sample and varnish colors of the Alkyd A and C samples were brown and the drying values were 10.7 and 6.2 h, respectively. In particular, it was determined that Alkyd A was slightly soluble at room temperature and soluble in all solvents except water. The discovered bio-based alkyd resin’s physical and chemical properties, as well as performance indexes, revealed that they have acceptable drying, chemical, and mechanical capabilities [105].

In the production of the alkyd resin, Hulsbosch et al. employed glutamic acid and its N-acrylated and N-alkylated derivatives [62]. According to the characterization results, alkyd resins with different molecular weights (g/mol) and the viscosity values (Pa s) were obtained and all results are summarized in Table S12. The degree of polymerization of the resins and their acids was evaluated by value. Accordingly, it has been stated that the value of the acid number of alkyd resins produced from glutamic acid is below 10 mg KOH/g according to industrial standards; therefore, most of the monomers in the diacid and fatty acids are involved in polycondensation. The oxidative thermal stability of alkyds was evaluated by thermogravimetric analysis (Figure S9). Accordingly, it was observed that most of the resin A and glutamic acid-based resins examined as references began to degrade slowly above 200 °C. It was stated that the decomposition accelerated with the increase in temperature and the resins completely degraded at 550 °C. When resins containing N-hexadecylglutamic acid dimethyl ester were compared to those containing N-almitoylglutamic acid dimethyl ester, resins containing N-hexadecylglutamic acid dimethyl ester were more stable.

In general, bio-based diacid monomers break down at a lower temperature than traditional diacids such as isophthalic acid. As a result, adding glutamic acid or its derivatives reduces the thermal stability of the resins.

Limited thermal stability is not an issue in areas where these resins will be utilized principally because typical industrial processes continue much below the decomposition temperature. Figure S10 depicts the results. In the side chain of resin H, there are no double bonds. Resin I is based on oleic acid and polymerizes to a dark color. Resin H, on the other hand, is practically colorless in

## 5. Conclusion

In this review, research on the use of bio-based chemicals that can be used instead of petroleum-derived chemicals in alkyd resin production has been examined in detail. Because of the scarcity of petroleum and petroleum derivatives, which are hazardous to human health and the environment, the development of bio-based, renewable, and sustainable chemicals, as well as their usage in the creation of polymers, has become critical in meeting future everyday demands. Since alkyds are inexpensive and less harmful than other resins, they are used in significant quantities especially in the paint industry. The increase in the use of bio-based chemicals in resins depends on the fact that they show properties close to those of the petroleum derivatives they are used instead. In addition, their prices are high due to the fact that their production is scarce worldwide and the need to research in which areas they will be used. It is critical to look at how the physical, thermal, and mechanical properties of resins vary when the percentage of bio-based compounds in alkyd resins rises, as this will affect the properties of the paint and coatings that result. As a future perspective, there will be a growing interest in the use of bio-based chemicals in every resin. The prices of petroleum and petroleum derivatives will increase day by day, and their production and usage areas will gradually decrease. For this reason, the increase in bio-based chemical production will cause a great complexity due to the need for agricultural lands, which are already limited. For the manufacturing of bio-based chemicals, forestry biomass should be used instead of agro-based biomass. Aquaculture is another raw material that can be useful to obtain the required biomass feedstock. With the increase in the use of biomass obtained from these sources in alkyd and similar resins, less harmfulness to the environment and human health can be achieved.

Bio-based chemicals with suitable functional groups such as fumaric acid, itaconic acid, aspartic acid, 3-hydroxy propionic acid, sorbitol, citric acid, furfural, succinic acid, malic acid can be used in the production of alkyd resins. The use of these chemicals as an alternative to petroleum-based chemicals is very valuable in alkyd resin production.

## Supplementary material

**Table S1:** Test results of the resins; Resin A, Resin B, Resin C, and Resin D [98].

Resin name	Weight average molecular weight (Mw)(g/mol)	Curing time at 210 °C (h)	Pencil hardness	Gloss (60°)
Resin A	2443	8	HB	85
Resin B	2649.5	7	HB	64.4
Resin C	2745	6	2B	48.2
Resin D	5596.5	4	2B	36.3

Figure S1 a) TGA and b) DSC thermograms of the resins [98].

**Table S2:** Test results of the resins for prepared with different materials and ratios [99].

Resin name	Hard drying time (min)	Pencil hardness	Acid value (mg KOH/g resin)	Gardner viscosity
A-LM-TMP/PE (50/50)	182	4H	15.7	Z6–Z7
A-SM-TMP/PE (50/50)	132	4H	4.7	Z7–Z8
LM-TMP/PE (50/50)	255	4H	19.2	> Z10
SM-TMP/PE (25/75)	370	4H	17.6	Z7–Z8
SM-TMP/PE (50/50)	390	2H	17.2	Z6–Z7
SM-TMP/PE (75/25)	150	2H	9.2	Z6–Z7
SM-TMP/PE (100/0)	240	4H	17.2	Z5–Z6

Figure S2 a) TGA curve of SIO-based alkyd resins, b) comparison of TGA curves of oil and fatty acid based alkyd resins, c) DTA curves of SIO-based alkyds, d) comparison of DTA curves of oil and fatty acid based alkyd resins [99].

**Table S3:** Test results of the REMESAR 1, CMESAR 2, CMESAR 3, CMESAR 4, SAP [60].

Resin name	Pencil hardness for scratch	Pencil hardness for gouge	Outdoor drying (min)	Indoor drying (min)
REMESAR 1	4H	4H	420	465
CMESAR 2	4H	4H	390	420
CMESAR 3	3H	3H	510	540
CMESAR 4	3H	3H	580	635
SAP	6H	5H	360	420

Figure S3 Procedure for alkyd resin RSO and SBO [79].

**Table S4:** Test results of the resins prepared with different ratios [79].

Resin name	Tack free time (min)	Drying time (min)	Iodine value (gI _2_/100 g)	Saponification values (mg/KOH)
100% RSO	129	238	157.90	216.21
100% SBO	131	241	142.00	228.61
80% RSO	132	237	152.44	203.67
70% RSO	128	235	149.90	171.04
50% RSO	130	237	143.16	161.97
30% RSO	132	239	142.26	142.89
10% RSO	133	343	142.41	135.62

**Table S5:** Test results of the resins prepared with different TESPIC ratios [101].

Resin name	Tg	Gloss (20°)	Gloss (60°)	Contact angle	Pencil hardness
TESLOA0	6.4 ± 1.8	101.9 ± 3.1	111.3 ± 0.6	80.7 ± 1.1	4B
TESLOA10	7.5 ± 1.2	104.9 ± 1.2	111.3 ± 0.3	101.1 ± 0.6	4B
TESLOA20	5.9 ± 1.1	103.6 ± 3.0	111.0 ± 0.4	101.5 ± 0.8	3B
TESLOA30	7.4 ± 1.7	105.1 ± 1.1	112.2 ± 0.5	101.7 ± 0.5	3B
TESLOA40	4.0 ± 1.5	104.7 ± 1.8	112.9 ± 0.3	100.8 ± 0.4	2B

Figure S4 a) TG and b) DTC spectrums of tung oil-based acrylated alkyd resin with different content ((a) 0 wt% IBOA, (b) 10 wt% IBOA, (c) 20 wt% IBOA, (d) 25 wt% IBOA, and (e) 30 wt% IBOA) [102].

**Table S6:** Test results of the resins prepared with different IBOA ratios [102].

Resin name	Viscosity (Pa s)	Water absorption (%)	Surface roughness (nm)	Pencil hardness
0 IBOA	7.94	3.54	1.02	H
10 IBOA	8.21	2.38	2.19	H
20 IBOA	8.09	1.06	3.92	2H
25 IBOA	8.16	0.99	4.39	3H
30 IBOA	8.44	0.89	5.26	3H

**Table S7:** Test results of the resins prepared with different monomer ratios [103].

Resin name	Gardner viscosity	Color	Dry-hard time (min)	Pencil hardness
SS-0%	Z5	12	160	4H
LS-0%	Z4	13	150	4H
SS-2%	Z5	7	130	2H
SS-4%	Z4	7	120	2H
SM-0%	Z10	10	240	2H
LM-0%	Z10	3	230	2H
SM-2%	Z10	3	180	HB
SM-4%	Z9	3	160	HB

Figure S5 a) TGA thermograms of SS-0%, LS-0%, SM-0%, LM-0%, SM-0%, SM-2%, and SM-4% [103].

**Table S8:** Test results of the resins that has different iodine values [105].

Resin name	Iodine value (I _2_/100 g)	Saponification value (mg/KOH g)	Acid value (mg/KOH g)	Specific gravity	Refractive index (30 ºC)
LASOMAR I	72.1 ± 0.74	205.2	6.34	1.91	1.500
LASOMAR II	78.1 ± 1.05	295.8	10.37	1.18	1.494
LASOMAR III	83.2 ± 1.0	332.5	7.85	1.72	1.491

**Table S9:** Test results of the resins prepared with different formulations [106].

Resin name	Water resistance (cold, 12 days)	Alkali resistance (0.1 M NaOH, 12 h)	Acid resistance (0.1 M H _2_SO _4_, 12 days)	Salt resistance (5%, w/w, NaCl, 12 days)
Alkyd-A	4	2	5	5
Alkyd-B	5	1	3	4
Alkyd-C	5	2	5	5
Alkyd-D	5	1	3	5

(0, completely removed; 1, cracked and partially removed; 2, partially cracked; 3, loss of gloss; 4, slight loss of gloss and 5, practically unaffected)

**Table S10:** Test results of the resins EA, EA1, EA2, EA3 and EA4 [50].

Resin name	Gloss (60°)	Pencil hardness	Scratch hardness	Td5 (°C)	Td30 (°C)
EA	95.24	3H	1950	213.29	347.25
EA1	98.42	3H	2000	228.18	351.78
EA2	101.2	4H	2500	233.93	356.38
EA3	102.3	4H	2100	226.92	359.32
EA4	108.20	5H	2250	246.97	371.12

Figure S6 a) DSC and b) TGA thermograms of EA, EA1, EA2, EA3, and EA4 [50].

Figure S7 Photos of different resin coatings before and after 500 h corrosion test in 3.5% NaCl solution [50].

Figure S8 Compound (a) is a bio-based alternative for petro-based rigid dicarboxylic acids, whereas compounds (b) and (c) are renewable alternatives for benzoic acid (R = H for glycine; R = benzyl for phenylalanine) [116].

**Table S11:** Test results of the resins; Resin 1, Resin 2, and Resin 3 [116].

Resin name	König hardness (s)	Drying tack-free time (h:min)
Resin 1	76	0:34
Resin 2	83	0:31
Resin 3	89	1:38

**Table S12:** Test results of the resins from A to I [105].

Resin name	Molecular weight (g/mol)	Viscosity value (Pa s)
A	2573	1.2
B	1734	1.0
C	1762	0.34
D	4070	2.4
E	1923	0.043
F	n.d.	n.d.
G	1660	0.092
H	1630	solid
I	n.d.	solid

Figure S9 Thermogravimetric curves of certain alkyd resins and N-palmitoylglutamic acid dimethyl ester monomer [105].

Figure S10 Comparison of studied resins A–I for color [105].

## Figures and Tables

**Table 1:** Iodine values of some oils.

Type of oil	Iodine value
Canola	110–120
Coconut	6–11
Corn	102–130
Olive	79–88
Peanut	84–100
Sunflower	110–143
Sesame	103–116
Soy bean	120–143
Palm	4–22
Rapeseed	97–108
Jatropha	105
Kenaf	86
Mustard	98–110
Ghee	26–38

**Table 2:** Chemical structures and natural appearance of some plant-based oils for alkyd resins.

Oil	Chemical	Alkyd resin sample
**Linseed oil**	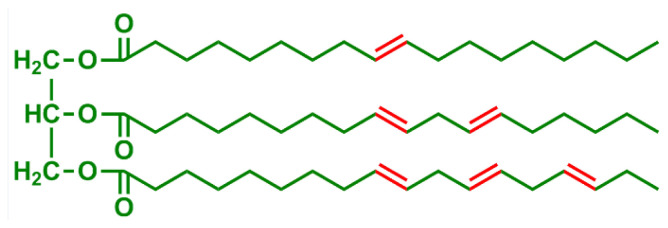	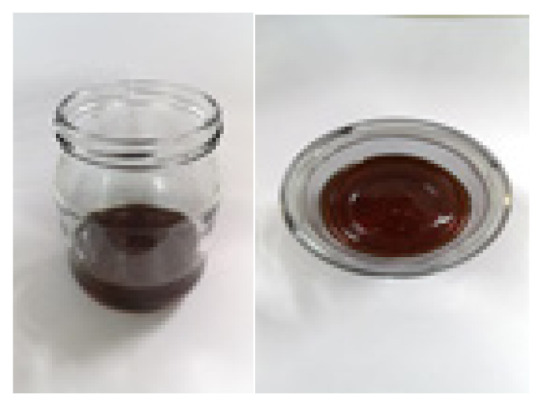
**Soybean oil**	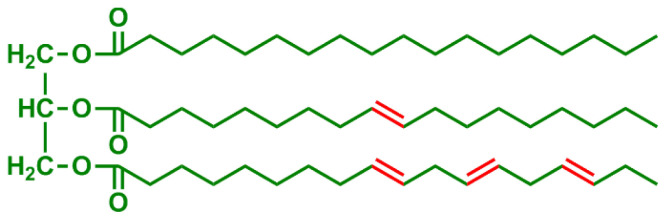	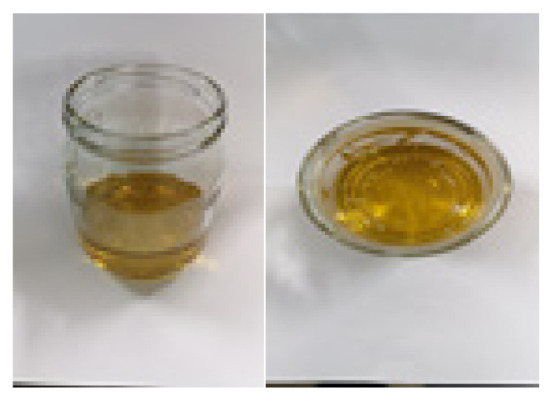
**Sunflower oil**	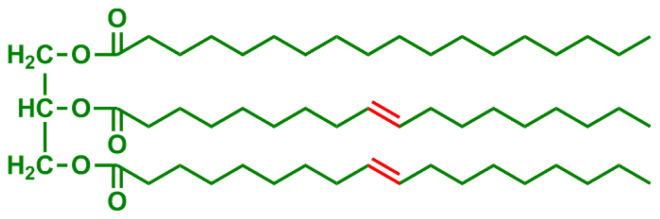	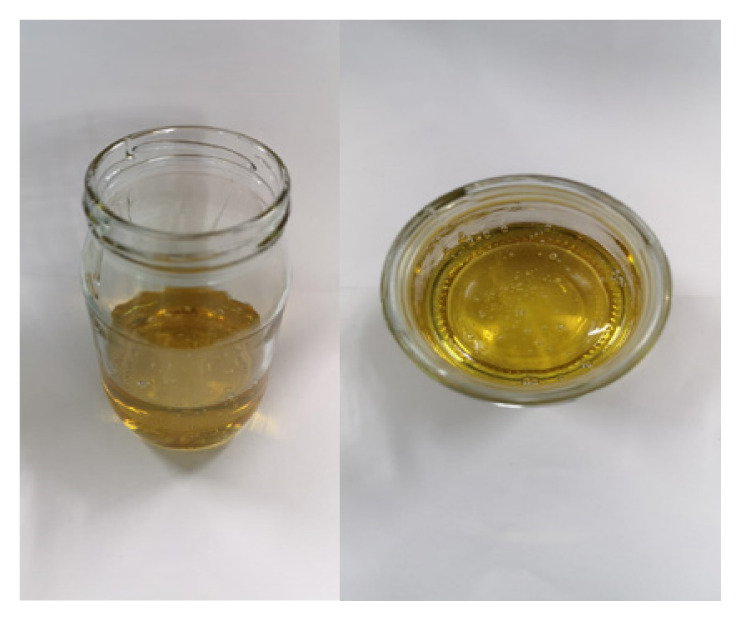
**Tall oil**	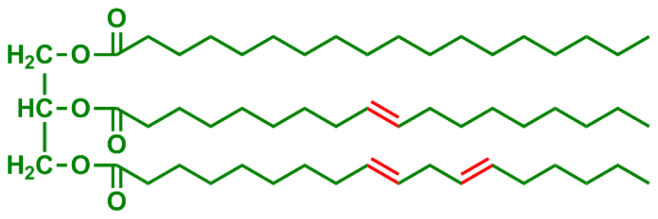	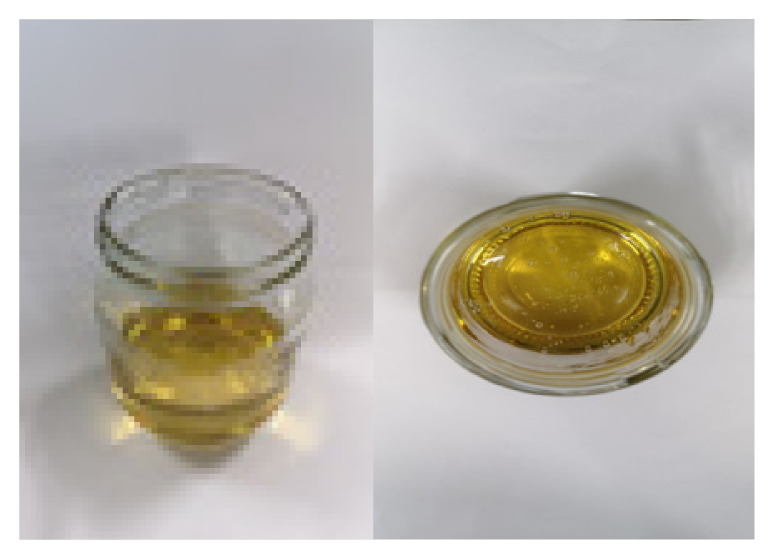
**Coconut oil**	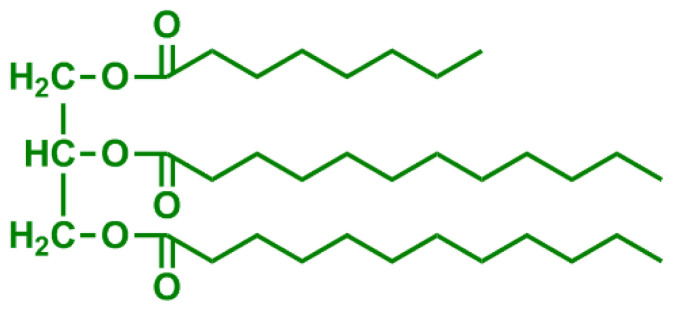	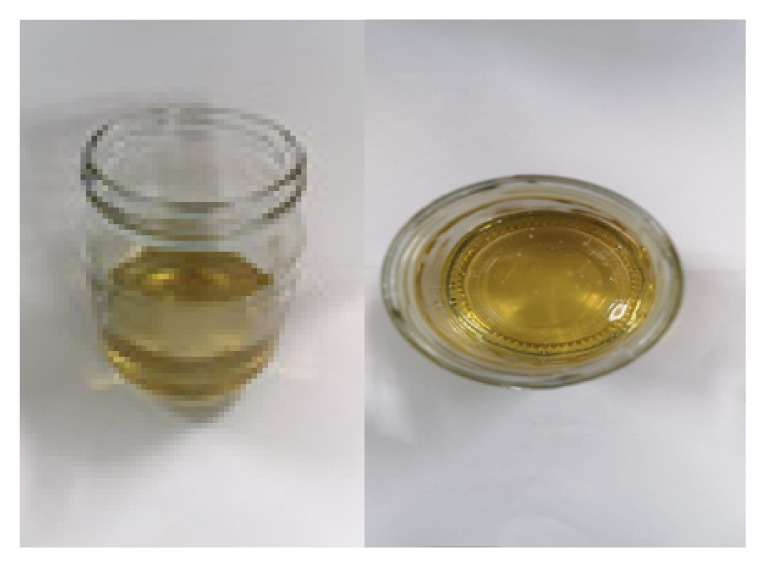
**Castor Oil**	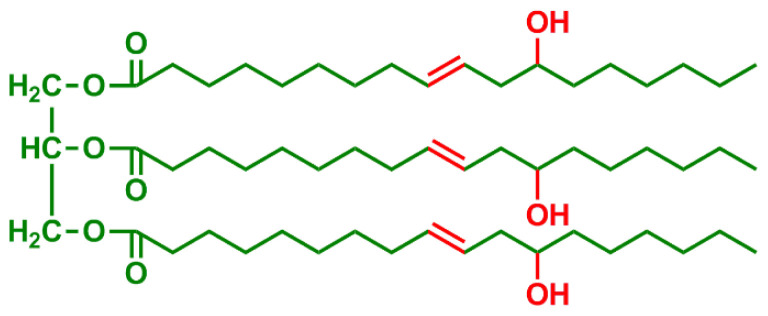	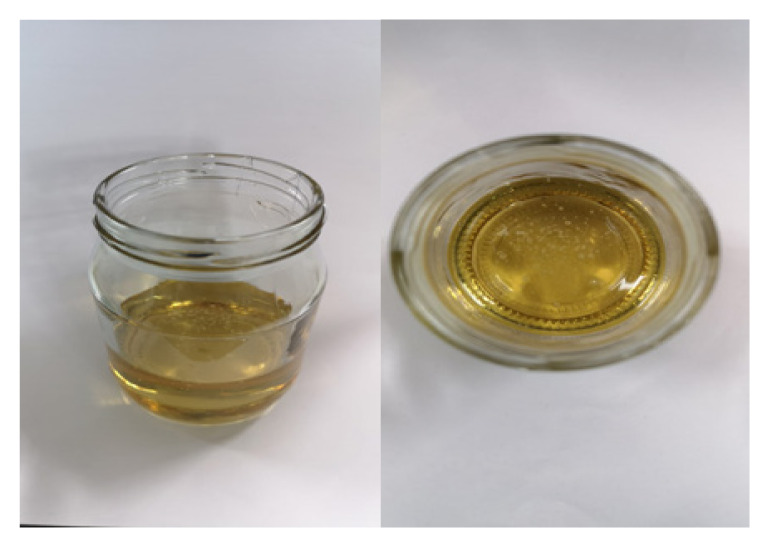

**Table 3:** Some application areas of long oil alkyds.

Metal anticorrosive primers	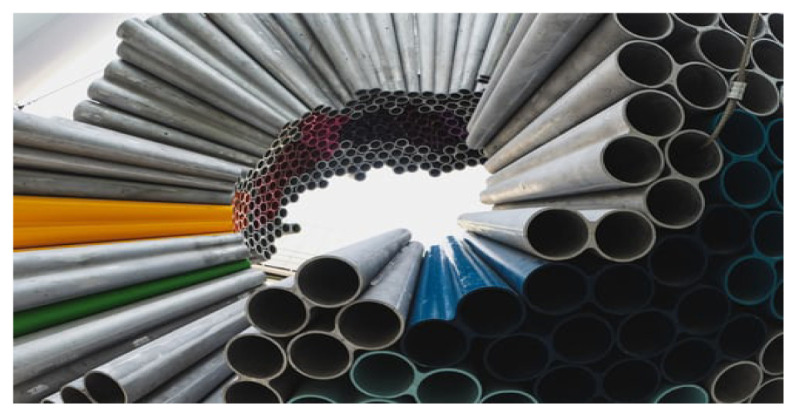	[61,63]
Wood varnishes	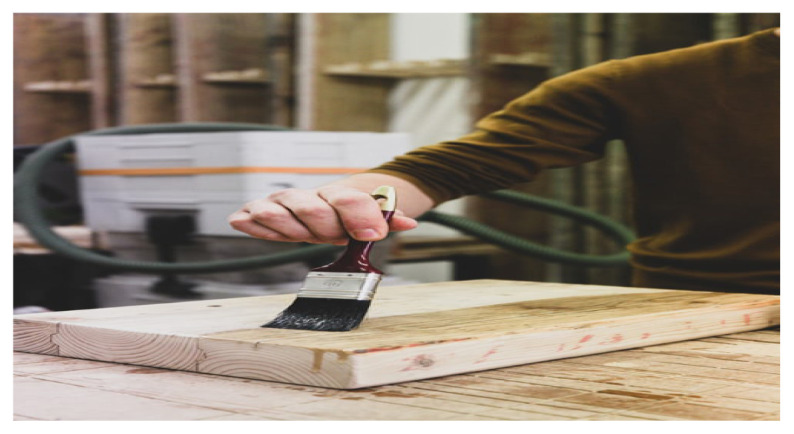	[4,5,64]
Architectural coatings	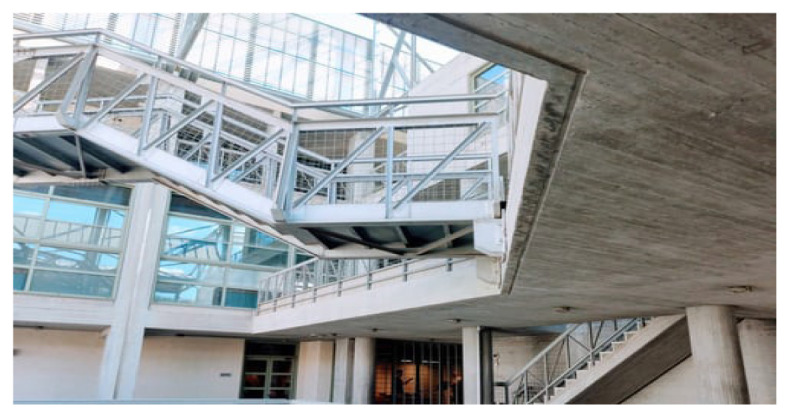	[65]
Indoor and outdoor paints	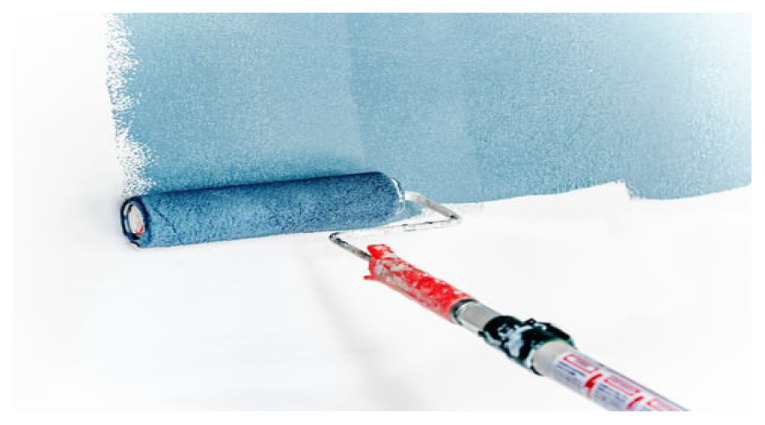	[60,66,67]

**Table 4:** Some application areas of medium oil alkyds.

Industrial coatings	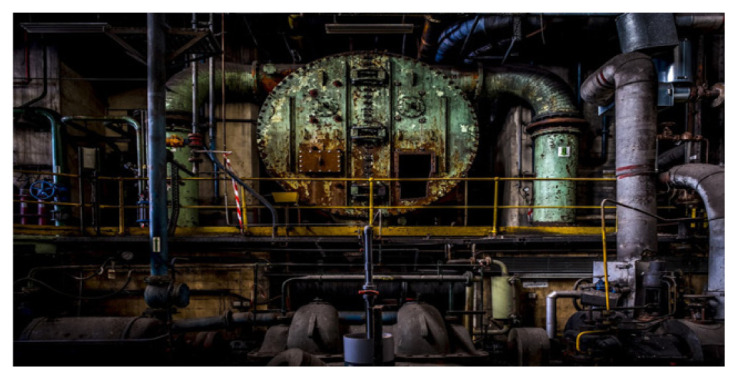	[68–71]
Industrial rapid paints	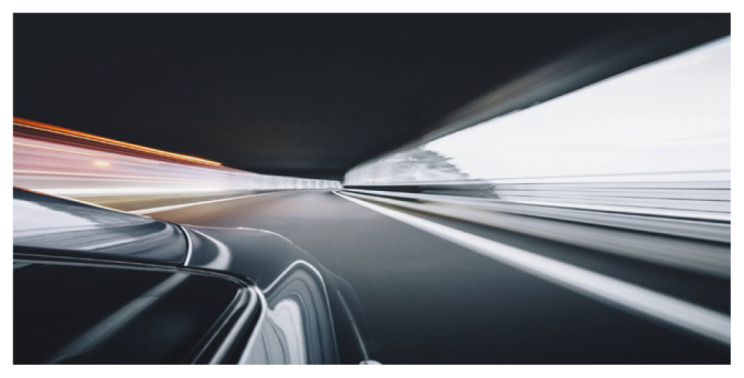	[72]
Wood surfaces	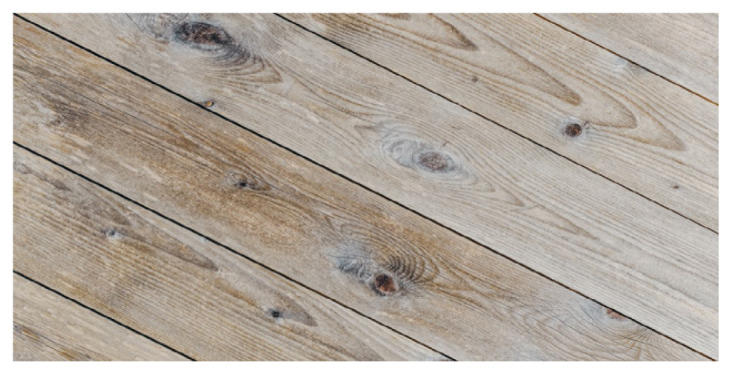	[60,73–75]
Rood marking and floorings	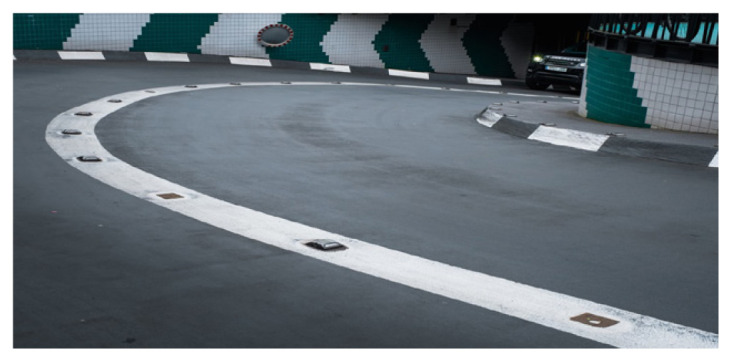	[76]
Architectural coatings	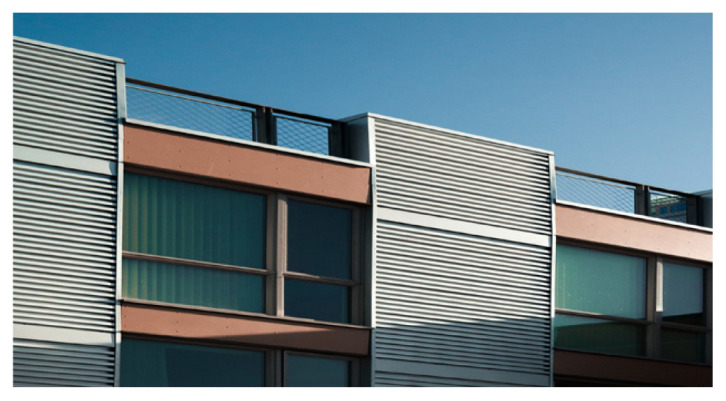	[77]

**Table 5:** Some application areas of short oil alkyds.

Manufacturing industry	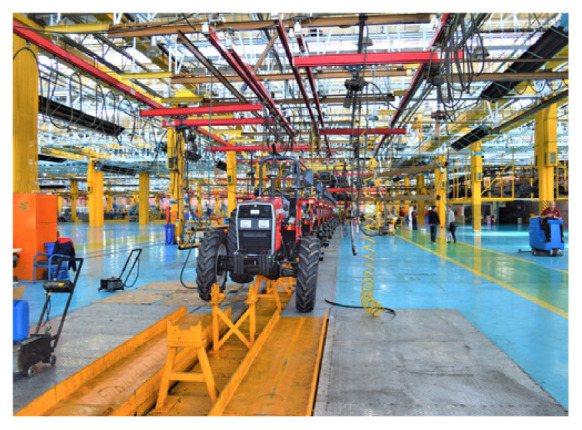	[15]
Industrial rapid paints	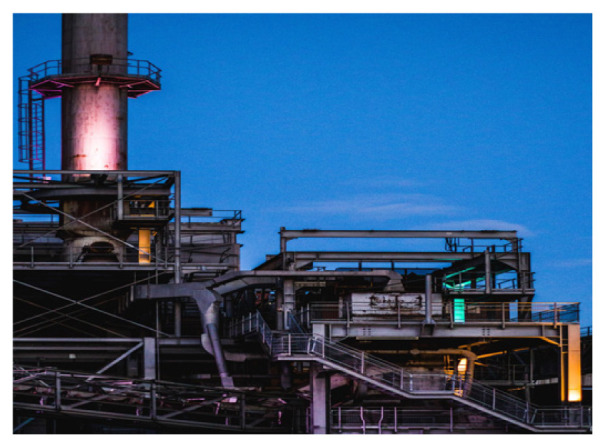	[65]
Wood surfaces	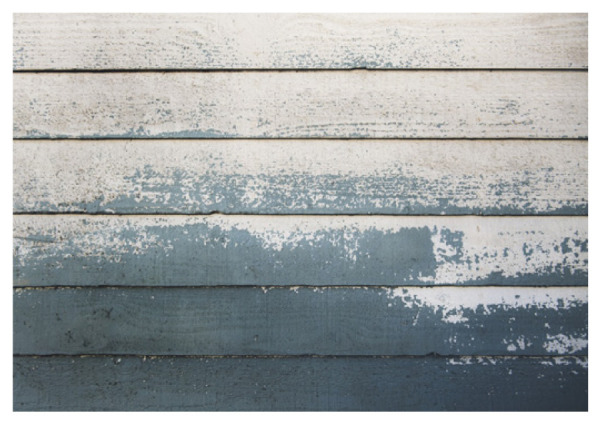	[78]
Architectural coatings	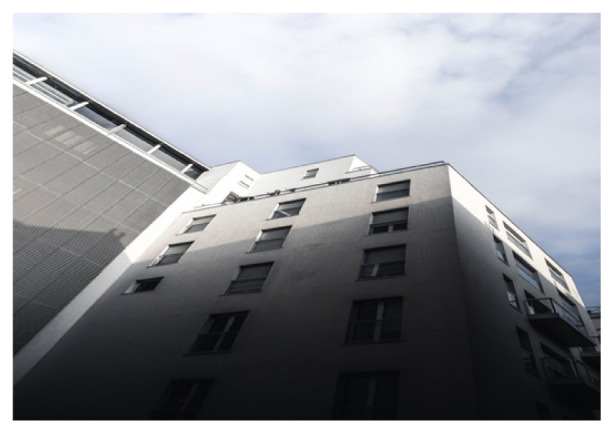	[79]

**Table 6:** Some application areas of urethane alkyds.

Marine applications	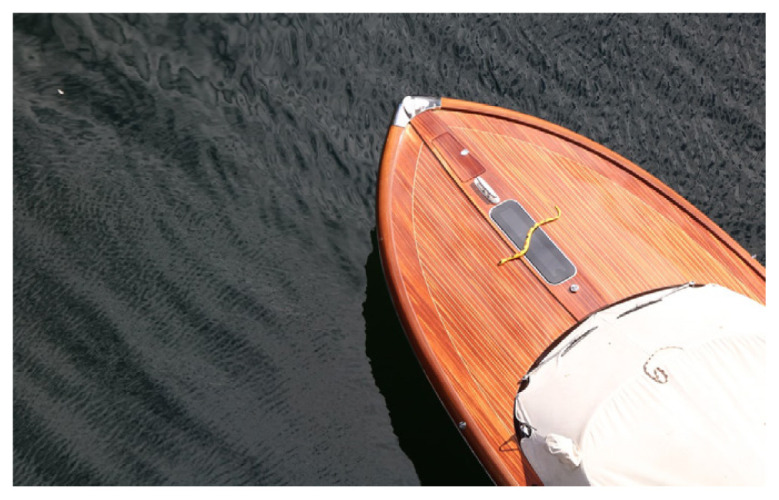	[81]
Anticorrosive for marine paints	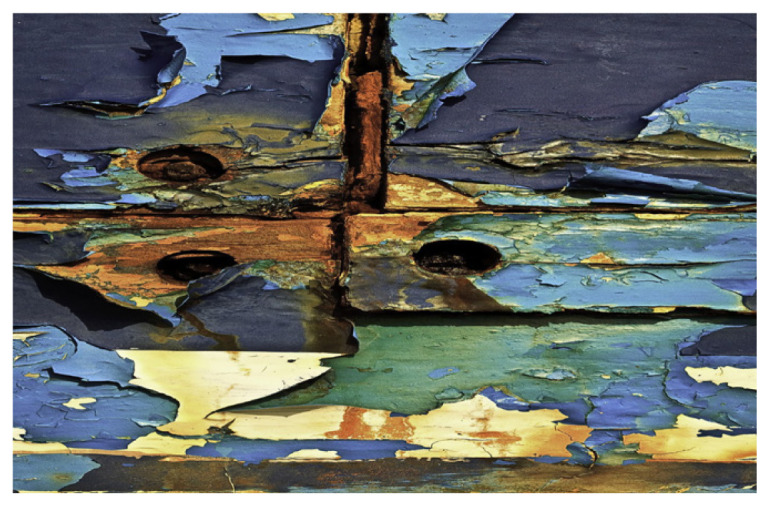	[82–84]
Wood varnishes	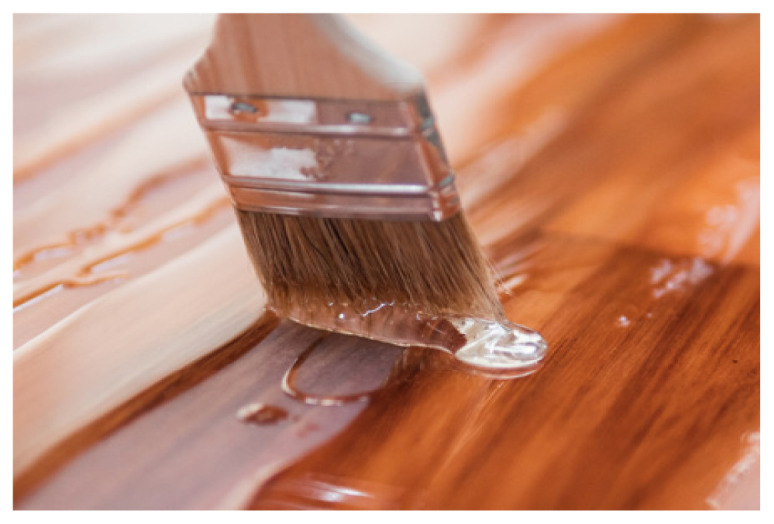	[85]

**Table 7:** Some application areas of chain-stopped alkyds.

Industrial paints	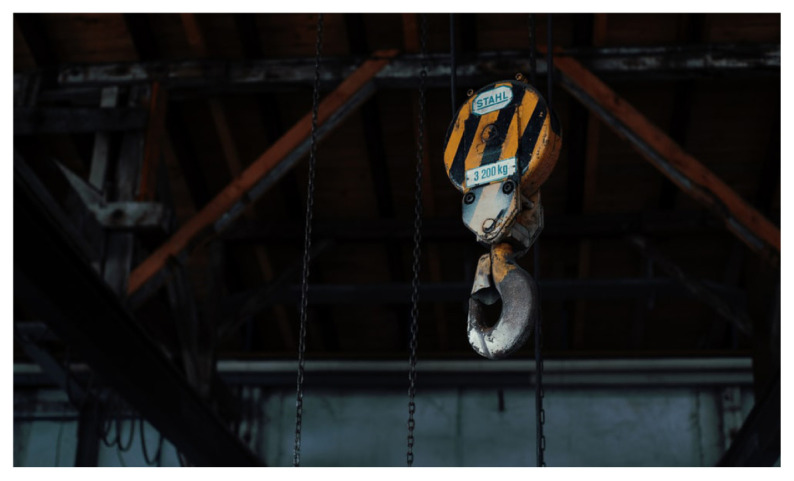	[87,88]
Anticorrosive varnishes	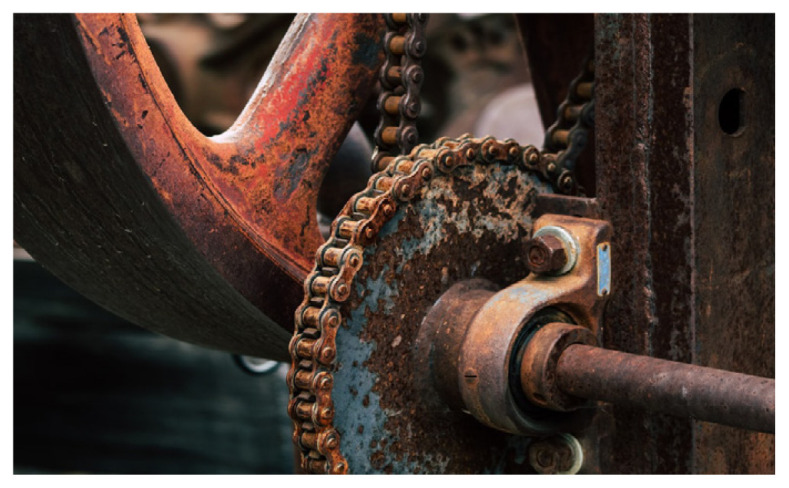	[24,82]
Wood surfaces	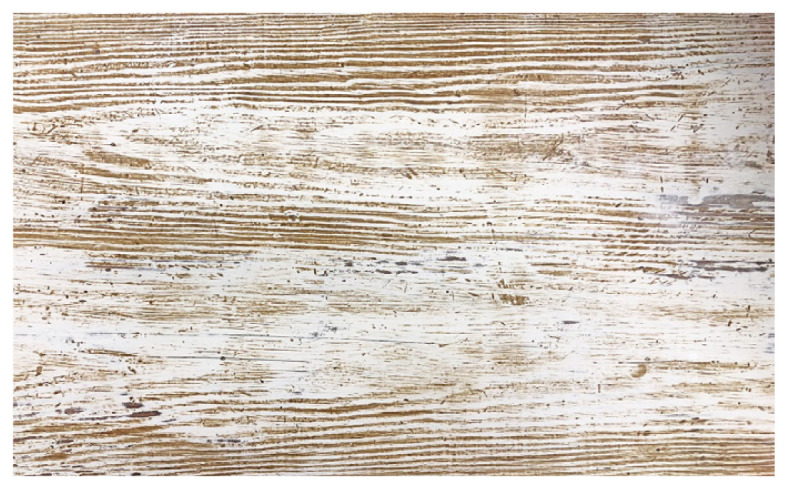	[89]
Hammerton	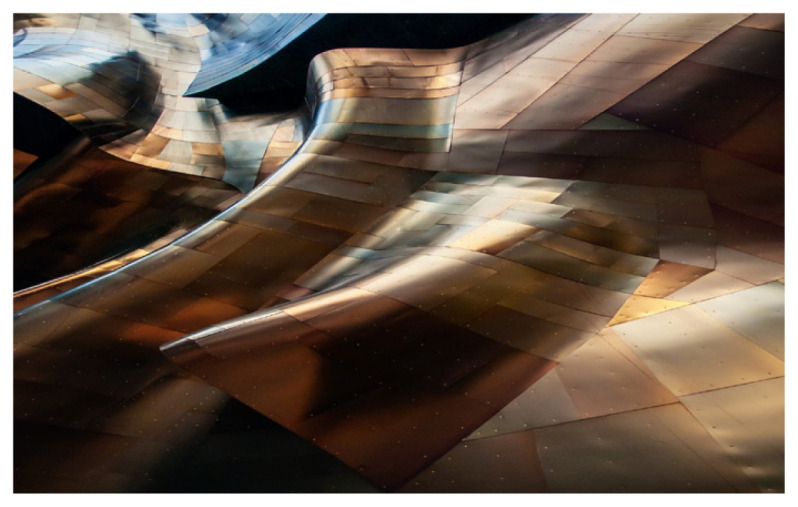	[90]

**Table 8:** Some application areas of polyurethane alkyds.

Wood surfaces	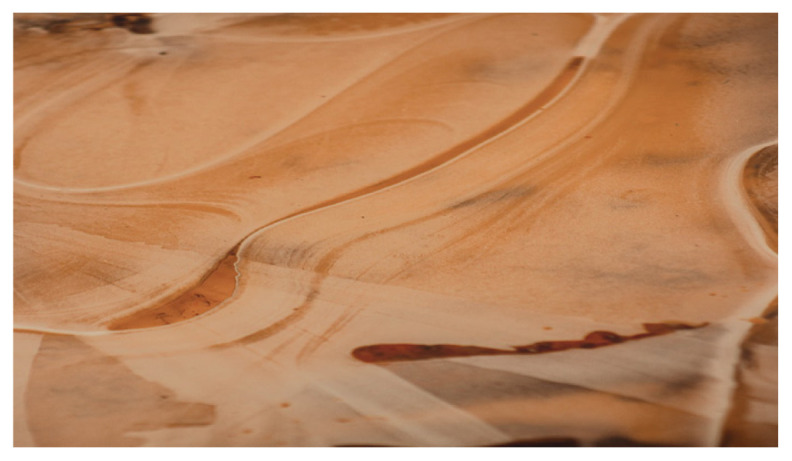	[91–93]
Metal surfaces	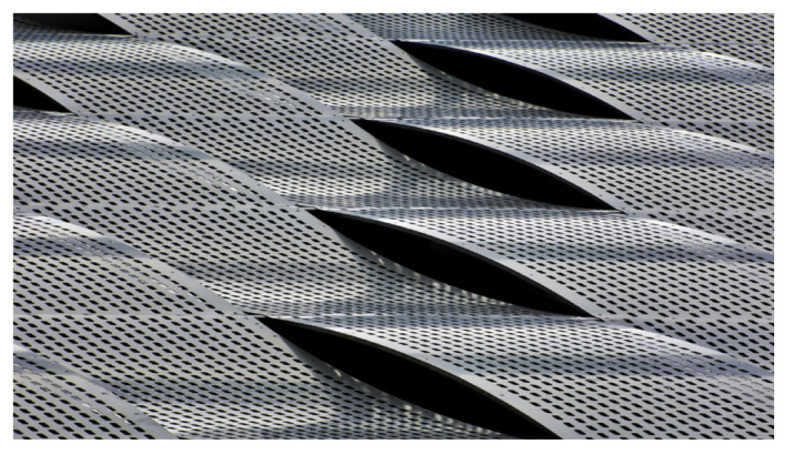	[94,95]

**Table 9:** Some petroleum-based alkyd synthesis monomers and compositions of the resins given in the literature.

Resin Name	Component I	Component II	Component III	Component IV	Reference
	***Oil (g)***	***Maleic Anhydride (g)***	***Phthalic Anhydride (g)***	***Glycerol (g)***	[98]
Resin A	38.68 (Neem seed oil)	0	17.772	7.36	
Resin B	38.68 (Neem seed oil)	2.941	13.330	7.36	
Resin C	38.68 (Neem seed oil)	5.883	8.887	7.36	
Resin D	34.53 (Neem seed oil)	7.876	3.965	7.36	
	***Oil (%)***	***Trimethylolpropane (%)***	***Pentaerythritol (%)***	***Phthalic anhydride (%)***	[99]
A-LM-TMP/PE (50/50)	53.9 (Fatty acid)	9.2	9.2	27.5	
A-SM-TMP/PE (50/50)	53.9 (Fatty acid)	9.2	9.2	27.5	
LM-TMP/PE (50/50)	53.9 (Linseed oil)	9.2	9.2	27.5	
SM-TMP/PE (25/75)	53.9 (Sacha inchi oil)	4.6	13.8	27.5	
SM-TMP/PE (50/50)	53.9 (Sacha inchi oil)	9.2	9.2	27.5	
SM-TMP/PE (75/25)	53.9 (Sacha inchi oil)	13.8	4.6	27.5	
SM-TMP/PE (100/0)	53.9 (Sacha inchi oil)	18.4	0	27.5	
	***Oil (%)***	***Trimethylolpropane (%)***	***Pentaerythritol (%)***	***Phthalic anhydride (%)***	[60]
REMESAR 1	60.00 (Refined melon seed oil)	30.60	0.08	59.40	
CMESAR 2	60.00 (Crude melon seed oil)	30.60	0.08	59.40	
CMESAR 3	75.00 (Crude melon seed oil)	21.92	0.03	53.07	
CMESAR 4	90.00 (Crude melon seed oil)	20.34	0.03	39.66	
	***Oil (kg/m*** *^3^* ***)***	***Maleic anhydride (%)***	***Rosin (%)***	***Glycerol (%)***	[100]
NAR-I	40.00 (Coconut oil)	7.50	42.50	10.00	
	***Oil (g)***	***Phthalic Anhydride (g)***	***Glycerol (g)***		[79]
100% RSO	503.61 (Rubber seed oil)	195.21	301.18		
100% SBO	503.61 (Soybean oil)	195.21	301.18		
80% RSO	402.91 (Rubber seed oil) 100.73 (Soybean oil)	195.21	301.18		
70% RSO	351.55 (Rubber seed oil) 151.09 (Soybean oil)	195.21	301.18		
50% RSO	251.82 (Rubber seed oil) 251.82 (Soybean oil)	195.21	301.18		
30% RSO	151.03 (Rubber seed oil) 352.55 (Soybean oil)	195.21	301.18		
10% RSO	100.73 (Rubber seed oil) 409.91 (Soybean oil)	195.21	301.18		
	***Oil (g)***	***Phthalic Anhydride (g)***	***Glycerol (g)***		[101]
LOA	170.00 (Linseed Oil)	80.00	45.00		
	***Oil (g)***	***Phthalic Anhydride (g)***	***Glycerol + LiOH (g)***		[102]
Acrylated alkyd resin	19.61 (Tung oil)	19.25	10.13		
	***Oil (g)***	***Glycerol (g)***	***Phthalic Anhydride (g)***	***Maleic Anhydride (g)***	[103]
SS-0%	63.11 (Sacha inchi oil)	35.32	67.57	-	
LS-0%	63.11 (Linseed oil)	35.32	67.57	-	
SS-2%	63.13 (Sacha inchi oil)	35.68	65.85	1.34	
SS-4%	63.13 (Sacha inchi oil)	35.67	64.50	2.69	
SM-0%	86.03 (Sacha inchi oil)	25.06	54.91	-	
LM-0%	86.03 (Linseed oil)	25.06	54.91	-	
SM-2%	86.03 (Sacha inchi oil)	25.05	53.81	1.10	
SM-4%	86.04 (Sacha inchi oil)	25.21	52.56	2.19	
	***Oil (W%)***	***Phthalic Anhydride (W%)***	***Glycerin (W%)***	***Zirconium Octoate 18% (W%)***	[104]
Alkyd resin A	54.9 (Soybean Oil)	26.63	16.22	0.199	
	***Oil %)***	***Phthalic Anhydride (%)***	***Glycerol (%)***		[105]
LASOMAR I	40 (Luffa aegyptiaca seed oil)	36.31	23.69		
LASOMAR II	50 (Luffa aegyptiaca seed oil)	30.2	18.9		
LASOMAR III	60 (Luffa aegyptiaca seed oil)	24.09	15.91		
	***Oil (g)***	***Maleic Anhydride (g)***	***Phthalic Anhydride (g)***	***Glycerol (g)***	[106]
Alkyd-A	50 (Gmelina seed oil)	-	50	50	
Alkyd-B	50 (Gmelina seed oil)	-	50	50	
Alkyd-C	50 (Gmelina seed oil)	25	25	50	
Alkyd-D	50 (Gmelina seed oil)	35	15	50	

**Table 10:** Some bio-based alkyd synthesis monomers and quantities in the literature.

Resin name	Component I	Component II	Component III		Reference
	Oil (mol)	Itaconic acid (mol)	Glycerol (mol)		[50]
Alkyd resin	1 (Linseed oil)	1	2		
	Oil (g)	Phthalic anhydride (g)	Pentaerythritol (g)	Imide (g)	[112]
Succinimide-based alkyd	367 (Soybean fatty acids)	193	324 (bio-based)	200	
	Oil (g)	Glycerol (g)	Sorbitol (g)	Phthalic anhydride (g)	[106]
ALKYD A and C	110 (rubber seed oil)	20	30	50	
	Oil (wt %)	Diacid or derivative (+additive) (wt %)	Pentaerythritol (wt %)	Isophthalic acid wt (%)	[62]
A	72 (Soy bean oil fatty acids)	6 (1,2,3,6-Tetrahydrophthalic acid)	17	5	
B	72 (Soy bean oil fatty acids)	6 (Glutamic acid)	17	5	
C	72 (Soy bean oil fatty acids)	6 (N-Palmitoylglutamic acid dimethyl ester)	17	5	
D	72 (Soy bean oil fatty acids)	6 (N-Palmitoylglutamic acid dimethyl ester)	17	5	
E	72 (Soy bean oil fatty acids)	6 (N-Hexadecylglutamic acid dimethyl ester)	17	5	
F	72 (Soy bean oil fatty acids)	6 (N-Palmitoylglutamic acid dimethyl ester + butylated hydroxytoluene)	17	5	
G	72 (Soy bean oil fatty acids)	6 (N-Palmitoylpyroglutamic acid methyl ester)	17	5	
H	72 (Palmitic acid)	6 (N-Palmitoylglutamic acid dimethyl ester)	17	5	
I	72 (Oleic acid)	6 (N-Palmitoylglutamic acid dimethyl ester)	17	5	
