# Supplementary figures and images for: Alkyd resins produced from bio-based resources for more sustainable and environmentally friendly coating applications
Source: Turk J Chem. 2022 Oct 8;47(1):1–23. doi: 10.55730/1300-0527.3511 (PMC10507036; doi:10.55730/1300-0527.3511)

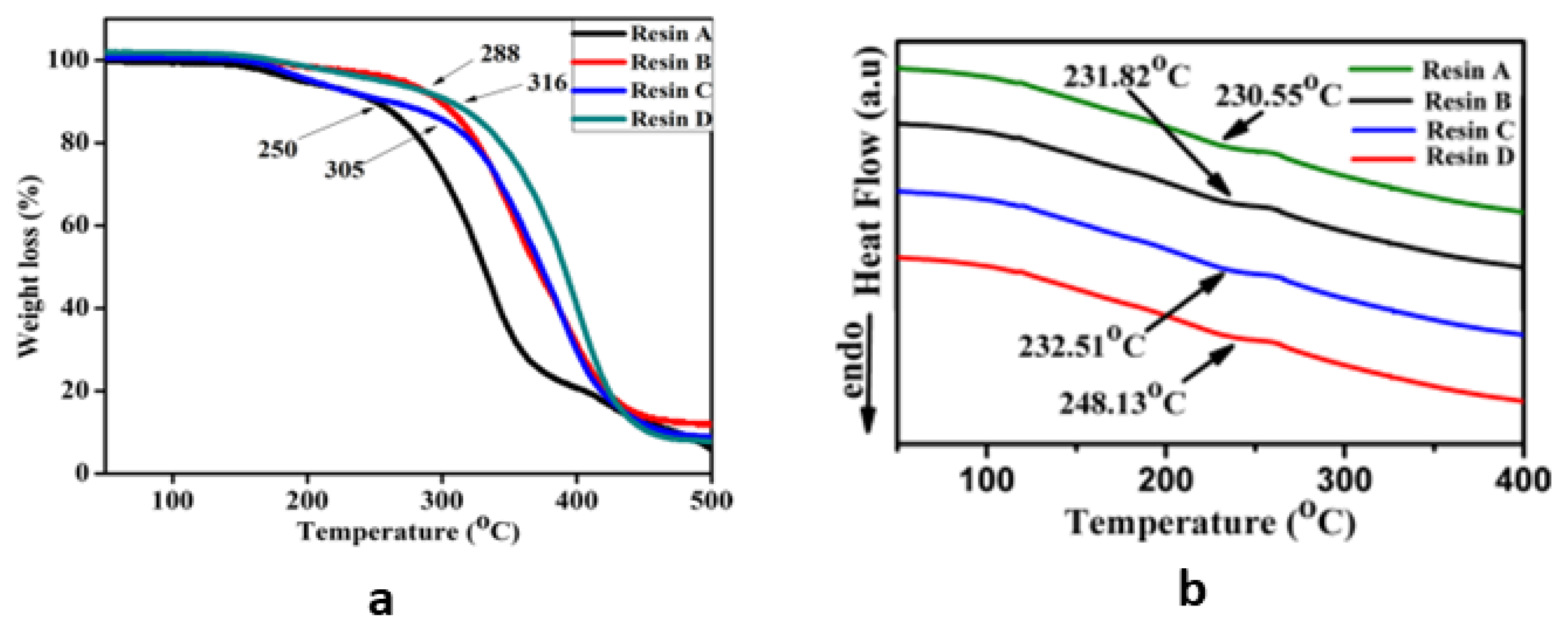

Supplement: Figure S1 — a) TGA and b) DSC thermograms of the resins [98]. [file turkjchem-47-1-1s1.tif]

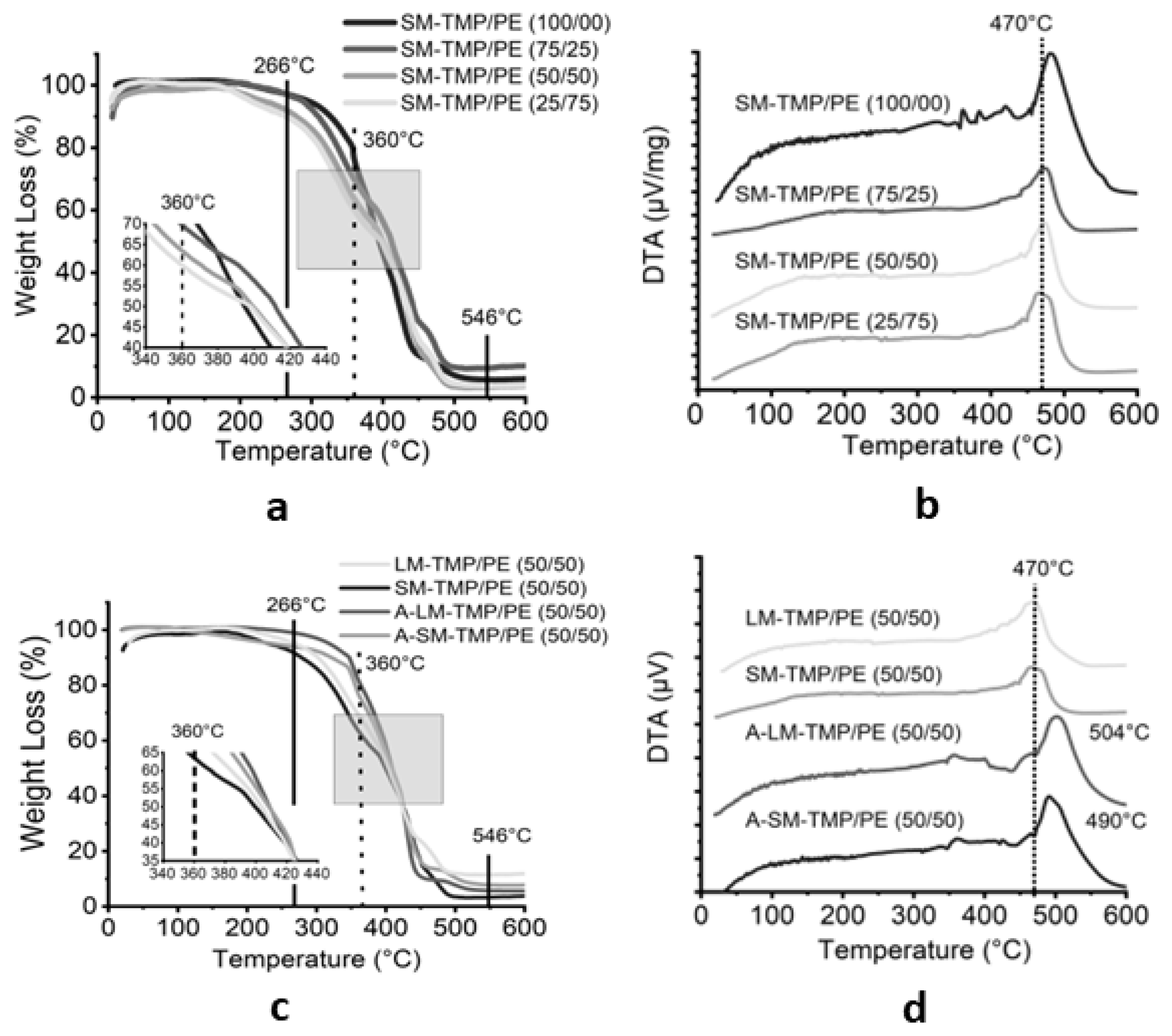

Supplement: Figure S2 — a) TGA curve of SIO-based alkyd resins, b) comparison of TGA curves of oil and fatty acid based alkyd resins, c) DTA curves of SIO-based alkyds, d) comparison of DTA curves of oil and fatty acid based alkyd resins [99]. [file turkjchem-47-1-1s2.tif]

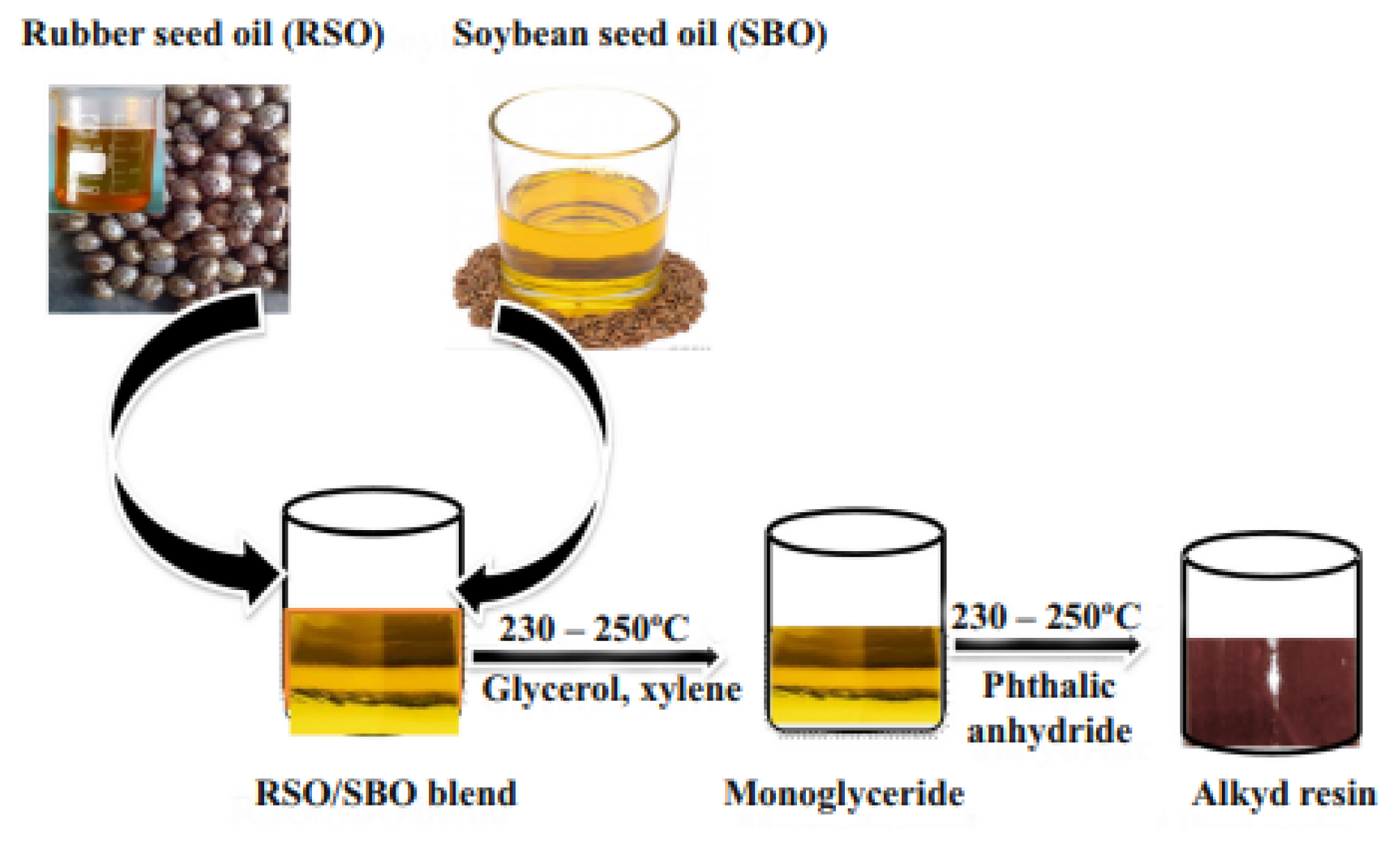

Supplement: Figure S3 — Procedure for alkyd resin RSO and SBO [79]. [file turkjchem-47-1-1s3.tif]

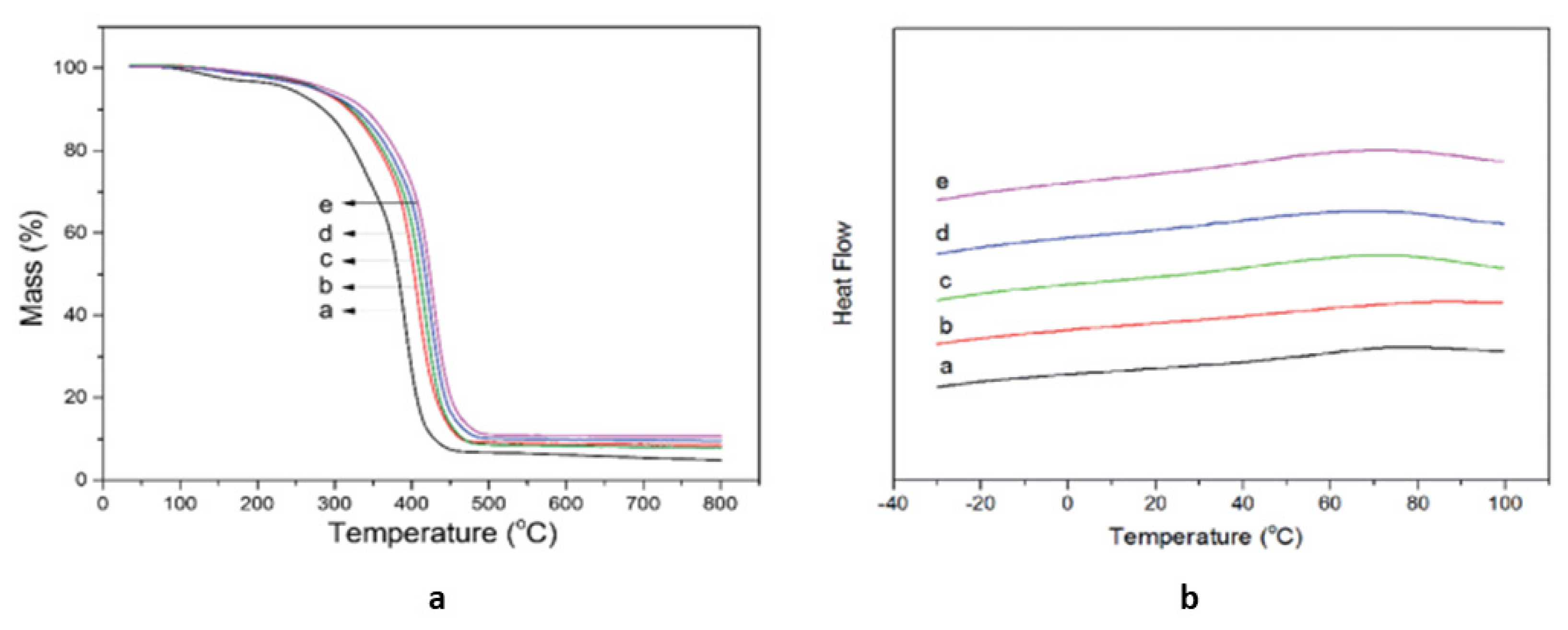

Supplement: Figure S4 — a) TG and b) DTC spectrums of tung oil-based acrylated alkyd resin with different content ((a) 0 wt% IBOA, (b) 10 wt% IBOA, (c) 20 wt% IBOA, (d) 25 wt% IBOA, and (e) 30 wt% IBOA) [102]. [file turkjchem-47-1-1s4.tif]

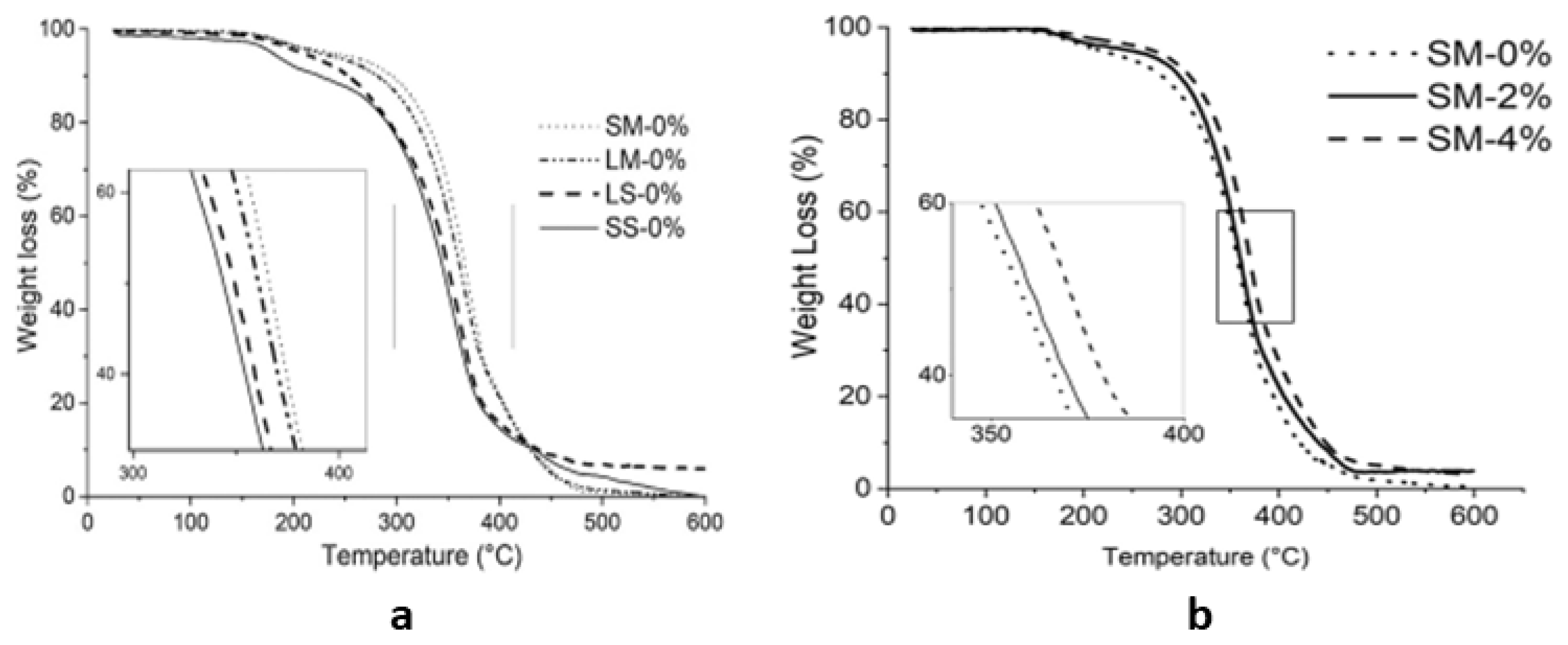

Supplement: Figure S5 — a) TGA thermograms of SS-0%, LS-0%, SM-0%, LM-0%, SM-0%, SM-2%, and SM-4% [103]. [file turkjchem-47-1-1s5.tif]

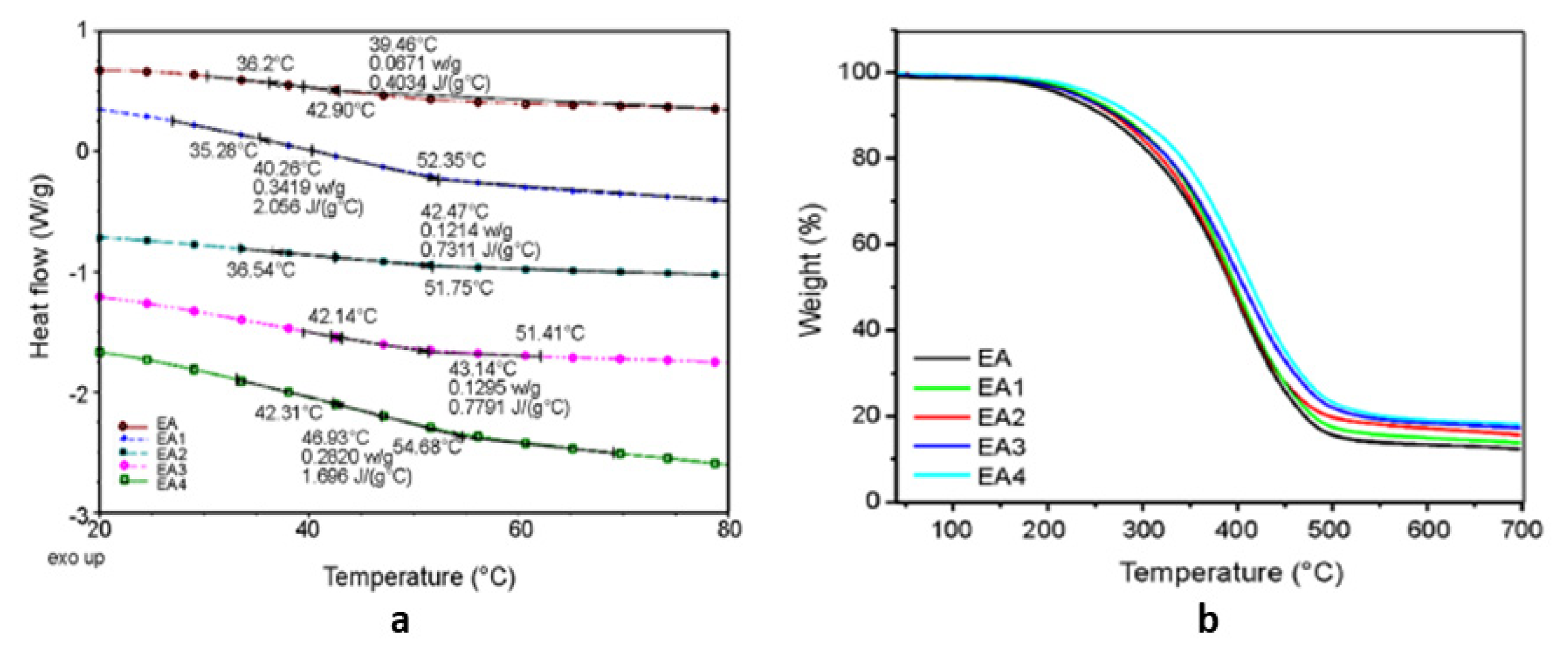

Supplement: Figure S6 — a) DSC and b) TGA thermograms of EA, EA1, EA2, EA3, and EA4 [50]. [file turkjchem-47-1-1s6.tif]

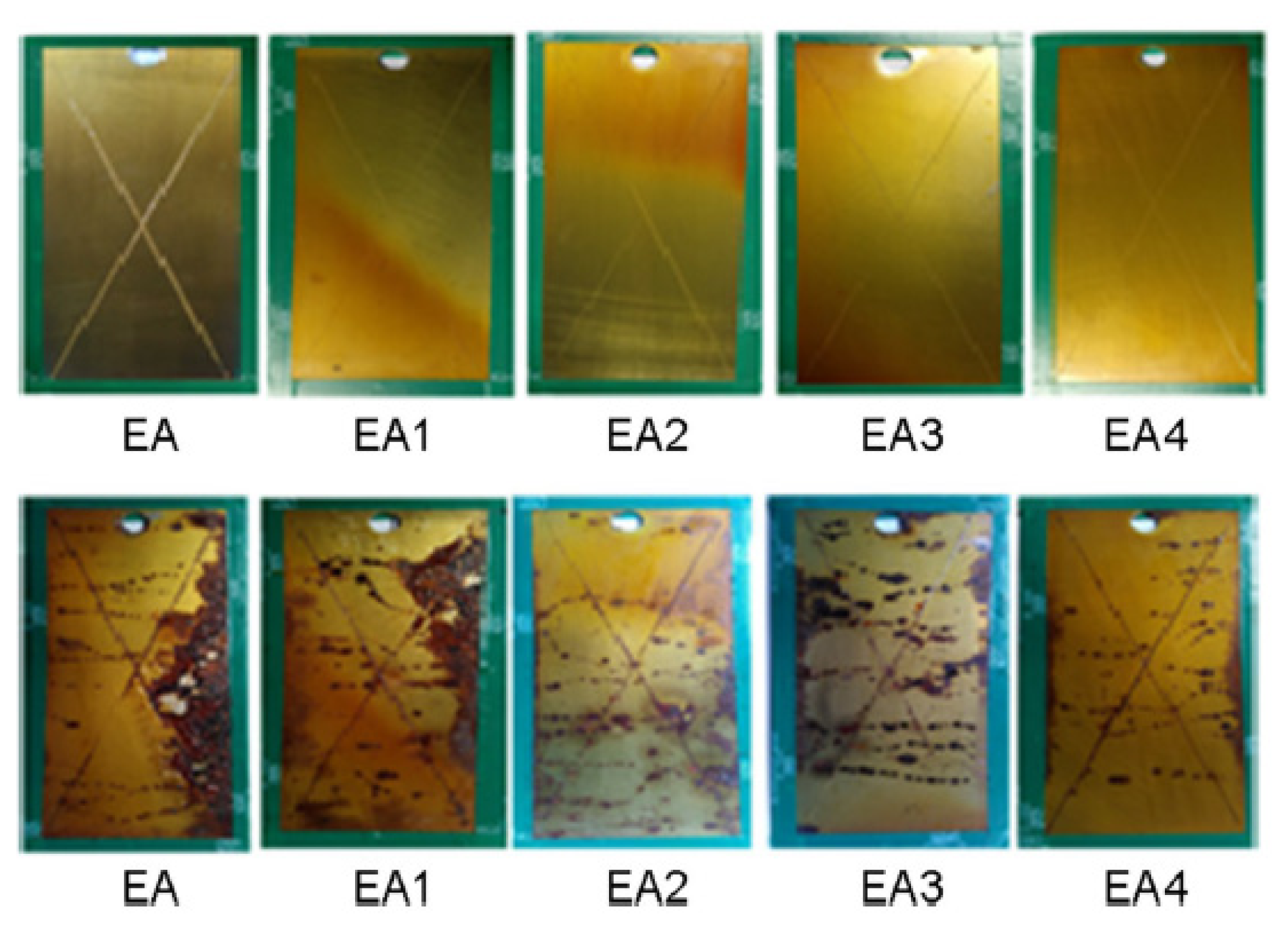

Supplement: Figure S7 — Photos of different resin coatings before and after 500 h corrosion test in 3.5% NaCl solution [50]. [file turkjchem-47-1-1s7.tif]

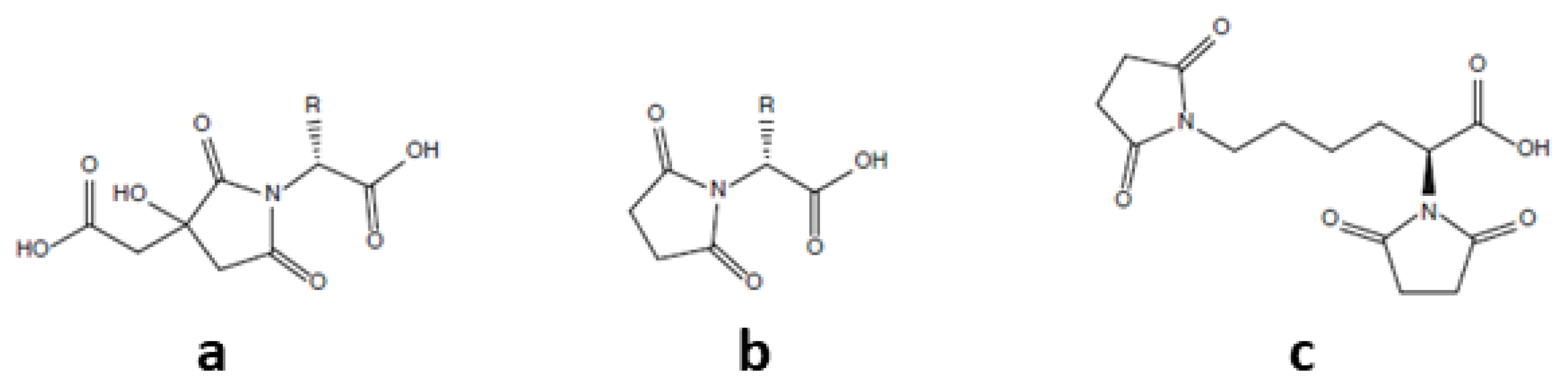

Supplement: Figure S8 — Compound (a) is a bio-based alternative for petro-based rigid dicarboxylic acids, whereas compounds (b) and (c) are renewable alternatives for benzoic acid (R = H for glycine; R = benzyl for phenylalanine) [116]. [file turkjchem-47-1-1s8.tif]

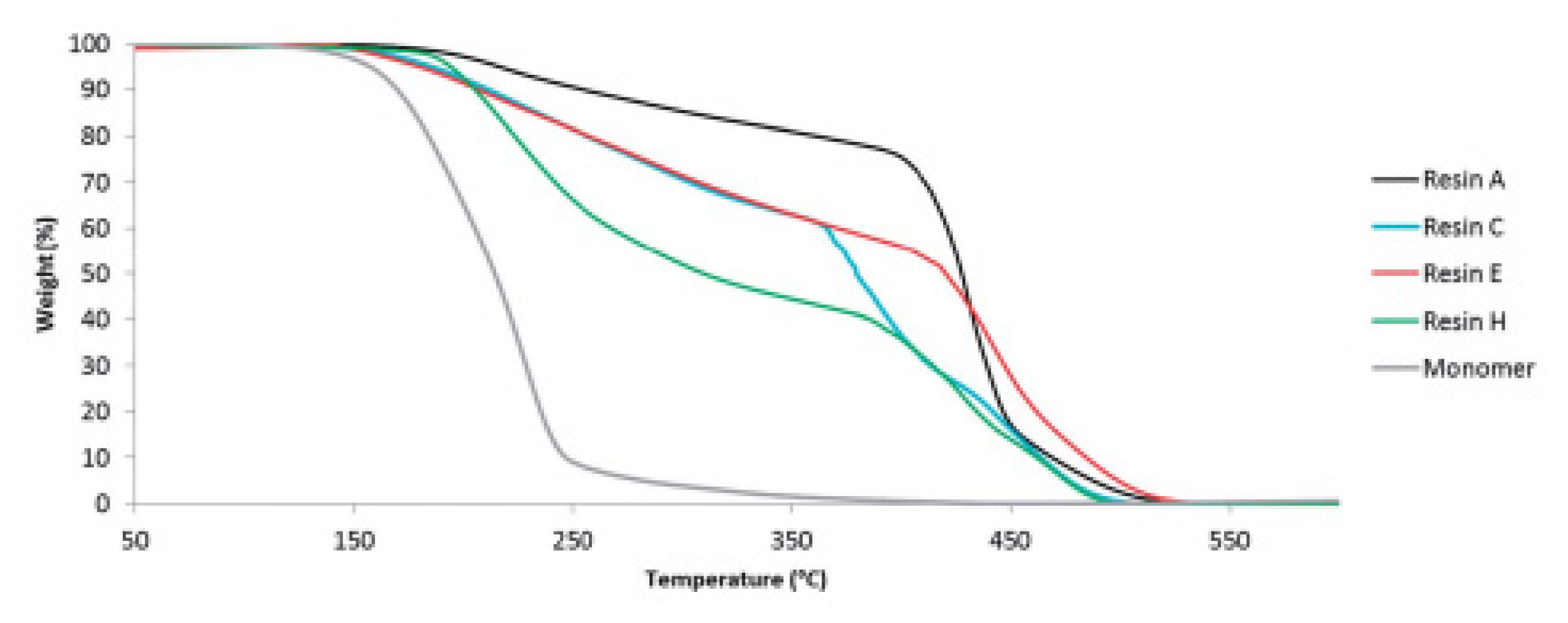

Supplement: Figure S9 — Thermogravimetric curves of certain alkyd resins and N-palmitoylglutamic acid dimethyl ester monomer [105]. [file turkjchem-47-1-1s9.tif]

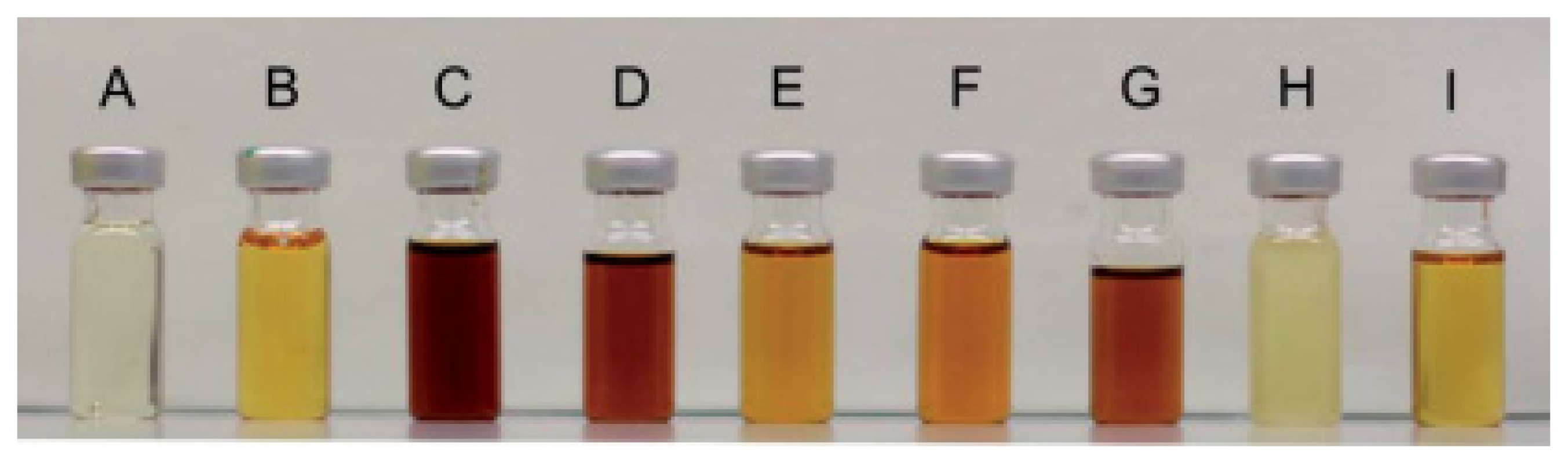

Supplement: Figure S10 — Comparison of studied resins A–I for color [105]. [file turkjchem-47-1-1s10.tif]
